# HoloMoA: a holography and deep learning tool for the identification of antimicrobial mechanisms of action and the detection of novel MoA

**DOI:** 10.3389/fmicb.2025.1640252

**Published:** 2025-08-22

**Authors:** Zohreh Sedaghat, Benoît Courbon, Héloïse Botrel, Hélène Dugua, Pawel Tulinski, Laethitia Alibaud, Lucia Pagani, Derry Mercer, Cyril Guyard, Christophe Védrine, Sophie Dixneuf

**Affiliations:** ^1^BIOASTER, Lyon, France; ^2^BIOASTER, Paris, France

**Keywords:** drug discovery, antimicrobial agents, mechanisms of action, holographic microscopy, deep learning, *Escherichia coli*

## Abstract

We propose an innovative technology to classify the Mechanism of Action (MoA) of antimicrobials and predict their novelty, called HoloMoA. Our rapid, robust, affordable and versatile tool is based on the combination of time-lapse Digital Inline Holographic Microscopy (DIHM) and Deep Learning (DL). In combination with hologram reconstruction. DIHM enables a label-free, time-resolved visualization of bacterial cell morphology and quantitative phase map to reveal phenotypic responses to antimicrobials, while DL techniques are powerful tools to extract discriminative features from image sequences and classify them. We assessed the performance of HoloMoA on *Escherichia coli* ATCC 25922 treated for up to 2 hours with 22 antibiotics representing 5 conventional functional classes (i.e. Cell Wall synthesis inhibitors, Cell Membrane synthesis inhibitors, Protein synthesis inhibitors, DNA and RNA synthesis inhibitors). First, using reconstructed phase images as input to a Convolutional Recurrent Neural Network (CRNN), we detected the MoA of known antibiotics with 95% accuracy. Secondly, we showed how our CRNN model combined with a Siamese Neural Network architecture can be used for the novelty assessment of the MoA of candidate antibiotics. We successfully evaluated our novelty detector on a test set containing three unseen molecules — two belonging to the conventional functional classes and one molecule from an additional class (Folate synthesis inhibitors, herein represented by trimethoprim-sulfamethoxazole). We demonstrated that the DIHM and DL combination provides a promising tool for determining the MoA of antimicrobial candidates using a large image database for known antimicrobials.

## Introduction

Antimicrobial resistance (AMR) was responsible for approximately 1.14 million directly attributable deaths in 2021 and was associated with approximately 4.7 million deaths the same year (GBD 2021 Antimicrobial Resistance Collaborators, 2024). The World Health Organization (WHO) ranked AMR as one of the top global public health threats and pointed out the lack of candidates in the antibiotics pipeline and limited access to conventional antibiotics, especially in LMICs (World Health Organization, 2024a, 2023). The WHO bacterial priority pathogens list ranks 15 families of antibiotic-resistant pathogens into three priority-level groups – critical, high and medium (World Health Organization, 2024b, 2017). The increasing number of Gram-negative pathogens resistant to last-resort antibiotics on this list underlines the need for novel antimicrobials. Novel antimicrobials should ideally not induce cross-resistance, and/or belong to a new chemical class, have new target(s) and new mechanisms of action (MoA)(World Health Organization, 2024a). Based on these criteria, WHO recently reported that, among the 13 new antibiotics that were approved since July 2017, only 3 belonged to classes with unknown resistance mechanisms. The current R&D pipeline, although innovative, is considered insufficient to combat AMR (World Health Organization, 2024a).

The mechanisms of action underlying existing antibiotic activities include inhibition of DNA replication, RNA synthesis, protein synthesis, cell wall synthesis, membrane functions, and the production of molecules such as folate or ATP (Kapoor et al., 2017). Regarding new antimicrobial agents, one of the bottlenecks following phenotypic screenings is identifying the MoA. There is no single universal standard to characterize MoA and its determination can be complex and time-consuming. Complete characterization often involves a combination of several technologies (Schäfer and Wenzel, 2020), making it expensive and resource heavy. A fast, robust, inexpensive and versatile tool, capable of detecting MoA novelty or even the main target of antimicrobial candidates would be very useful to ease the process of antibiotic MoA determination and accelerate the antimicrobial discovery pipeline.

Traditional methods for early-stage MoA classification are labor-intensive and costly. For example, macromolecular synthesis assays (MMS) are an ensemble of 5 assays which involve the incorporation of radiolabeled precursors into macromolecules of the different biosynthesis pathways – peptidoglycan (cell wall), DNA (replication), RNA (transcription), proteins (translation), fatty acids – to identify which pathway(s) is(are) inhibited (Cotsonas King and Wu, 2009). These assays suffer from low resolution, low accuracy, low throughput and show limitations for the identification of novel MoA (Nonejuie et al., 2013). There is a need for alternative assay/s that would be less expensive, safer (i.e., not requiring radiolabeled precursors), capable of processing not only antibiotics but also complex therapeutic solutions such as drug combinations, and capable of detecting novel MoA.

Recent methods use phenotypic assays that could be applied to library screening campaigns (Da Cunha et al., 2021), including dynamic metabolome profiling using time-of-flight mass spectrometry (Zampieri et al., 2018), transcriptomic (O’Rourke et al., 2020) and proteomic (Bandow et al., 2003) profiling. Optical methods and in particular imaging methods based on various microscopies are also available, and the accessibility of instrumentation and protocols as well as access to single bacterial cell resolution contributes to a gain in speed and cost (Nonejuie et al., 2013; Zoffmann et al., 2019; Martin et al., 2020; Ouyang et al., 2022; Zahir et al., 2019). MoA determination is based on morphological changes induced by different functional classes of antibiotics on a sensitive strain of a targeted bacterial species (Cushnie et al., 2016; Cylke et al., 2022). Among these methods, bacterial cytological profiling (BCP) is based on fluorescence labeling of DNA and cell wall followed by morphological analysis of each label after a specific period of incubation, either using an “optical sectioning” microscope and z-stack deconvolution (Nonejuie et al., 2013) or standard fluorescence images (Martin et al., 2020). A more recent implementation of label-based fluorescence imaging used non-cytotoxic labels to exploit the dynamic aspect of morphological changes and increase the resolution and accuracy of classification (Ouyang et al., 2022). Salgado et al. recently reviewed BCP implementations for various bacterial species and listed advantages and limitations of the technology (Salgado et al., 2025).

Label-free microscopy represents a further step forward in terms of simplification, cost reduction, safety and ecological considerations. No label means that live bacterial cells can be monitored during incubation with the antimicrobial agent, with the guarantee that nothing but the antimicrobial agent will modify the behavior of the cell. Phase contrast microscopy, for example, has been proposed for high-throughput, time-resolved, label-free screening of bacterial morphology to reveal phenotypic responses to antibiotics (Zahir et al., 2019). In the present work, we propose to evaluate an alternative label-free, robust and versatile technology for classifying the MoA of an antibacterial and detecting its novelty. The proposed technology, named HoloMoA, combines time-lapse digital inline holographic microscopy (DIHM) and deep learning (DL) analyses. DIHM is the simplest implementation of digital holographic microscopy (Popescu, 2011); it enables the revealing of absorption and phase modulations that light undergoes when probing the quasi-transparent bacteria. At 100 × magnification, the phase image of a bacterium translates to “thickness × refractive index” heterogeneity within the bacterial cell, without any fixation or staining. Based on the acquisition of out-of-focus images (i.e., holograms), DIHM does not rely on precise focusing mechanics (contrary to conventional microscopies) but rather on robust digital re-focalization. Here, we adapted and evolved an experimental approach, including devices and prototype, that had been set up during a previous diagnostic DIHM study, to perform antibiotic susceptibility testing of clinical *E. coli* isolates (Degoût-Charmette et al., 2025; Douet and Josso, 2017; Mahé et al., 2022).

Due to the abundance and high complexity of bacterial time-lapse images, their processing as well as the identification of the antimicrobial MoA were integrated within an automated pipeline. Increasingly sophisticated statistical methods have been developed to perform such classification. Recent breakthroughs in Machine Learning (ML) and Deep Learning (DL) showed great potential due to their ability to handle large image datasets and extract fine phenotypic information, sometimes not visible to the human eye, to predict a biological outcome of interest. Researchers have proposed a variety of approaches for the automated analysis of high content images to predict the MoA of drugs (Krentzel et al., 2023). Basic pipelines rely on extraction of morphological features (such as cell area, mean intensity, roughness…) from the images. This requires prior knowledge from the analyst and the use of image analysis software. The features can be combined to perform dimensionality reduction and distance-based clustering to detect MoA groups. Nonejuie et al. applied Principal Component Analysis on 14 features derived from ImageJ software followed by Euclidean distance clustering to highlight 5 MoA groups (Nonejuie et al., 2013). Martin et al. similarly computed 14 morphological features then applied MANOVA and single-linkage clustering to assess the novelty of the MoA of a drug candidate compared to 37 known antibiotics (Martin et al., 2020). Meanwhile, Ouyang et al. (2022) used features extracted from Fiji software (Schindelin et al., 2012) and set up a rule-based algorithm to assign antibiotics into 5 MoA classes. A limitation of the aforementioned basic pipelines is the need for prior expertise to compute the features, the presence of uninformative features susceptible to bring noise, and the inability to capture non-linear dependencies among the features. To address the latter aspect, some researchers applied a supervised ML classifier on top of pre-computed features to predict the MoA class of a drug. Zoffman et al. trained a Random Forest (RF) on over 100 features to classify 20 antibiotics among 6 MoA classes (Zoffmann et al., 2019). Likewise, Zahir et al. (2019) employed Soft Independent Modeling of Class Analogy (SIMCA) to predict 4 morphological groups while Simm et al. (2018) computed 842 features with CellProfiler (Carpenter et al., 2006) and trained different ML models (Bayesian Matrix Factorization, Random Forest, Deep Neural Network) to predict bioactivity of new compounds.

To overcome the drawback of depending on pre-computed features, end-to-end DL methods have been developed. They have shown great promise due to their ability to extract autonomously a huge number of discriminative features from unstructured data such as images and combine them to perform classification. 2-dimensional Convolutional Neural Networks (CNN2D) have been the most successful architecture to classify images (LeCun et al., 2015). CNN2D contain convolutional layers which learn automatically and adaptatively spatial features of the data at different scales, in a robust manner. CNN2D and their extensions have shown good performance for MoA prediction based on eukaryotic cell data. For instance, Kensert et al. (2019) used deep CNN2D pretrained on large, generic image datasets and fine-tuned them on a dataset of breast cancer cells exposed to 38 chemical compounds corresponding to 12 MoA and achieved high classification performance. Furthermore, Godinez et al. (2017) developed a multiscale CNN (M-CNN) that can analyze an image at 7 different resolutions in parallel and achieved cell morphology classification performance superior to existing deep CNN2D models. These methods are efficient to classify known MoAs but are either not designed to detect novel MoAs or deploy only a basic extension of their algorithm to address this question. A common approach is to measure distances in the feature space with respect to known classes. Still, the feature space may lack generalization to unknown data, especially if it is complex and high-dimensional (Chalapathy and Chawla, 2019). More specific approaches can be used to address novelty detection, such as One Class Support Vector Machines (Schölkopf et al., 2001), Auto-Encoders (Hasan et al., 2016) or Siamese Neural Networks (Zhou et al., 2021), but to our knowledge, they have not been used for drug screening yet.

In the present work, we propose a fully automated DL pipeline using CNN architecture for MoA classification, taking dynamic holographic data of *E. coli* exposed to bactericidal and bacteriostatic antibiotics as input. Moreover, our pipeline can be adapted for MoA novelty assessment of a drug candidate (i.e., novelty detector). We believe that this approach could be adapted to determine the MoA of antimicrobials against other pathogens. Our approaches introduce two main original aspects compared to state-of-the-art. First, we included a longitudinal component in our DL architecture as we tracked the structural and morphological changes of the bacteria over time. To consider this temporal dimension, we developed two extensions of the standard CNN architecture: 3-dimensional CNN (CNN3D) (Mahé et al., 2022) and Recurrent CNN (RCNN) (Barros et al., 2021). Second, we used specific DL architecture to provide a robust, quantitative assessment of the novelty of an antibiotic MoA. Specifically, we integrated CNN3D and RCNN into Siamese Neural networks (SNN), resulting in sCNN3D and sCRNN, trained to predict whether two antibiotics share the same MoA.

## Results

### Study design

We used *E. coli* ATCC 25922 which is recommended by the Clinical and Laboratory Standards Institute (CLSI) as a quality control strain for antimicrobial susceptibility tests (Clinical and Laboratory Standards Institute, 2024b). The holographic image dataset was built with 22 antibiotics split across 11 chemical subclasses and five functional classes constituting 5 “conventional” MoA: inhibition of DNA replication, inhibition of RNA synthesis, inhibition of protein synthesis, inhibition of cell wall biosynthesis, inhibition of membrane functions. Trimethoprim-sulfamethoxazole is a combination of folate synthesis inhibitors which was used herein to mimic an antibiotic candidate featuring a “novel” MoA to test the novelty detector; it represents a 6^th^ MoA class. The minimum inhibitory concentrations (MIC) obtained for all these molecules are listed in Supplementary Table S1. We generated the DIHM image dataset before testing the MoA classification tool and novelty detector. A single concentration of each compound was used to construct the final image dataset. This concentration was set at 1 × MIC, except for kanamycin, tobramycin, tetracycline and lymecycline, which started showing an effect at 2 × MIC, and trimethoprim-sulfamethoxazole at 8 × MIC (see Discussion). An untreated *E. coli* control was systematically tested for each new bacterial culture. Bacterial cultures and antibiotic exposures took place in Mueller Hinton broth (MHB), except for colistin which required Cation-Adjusted MHB (CAMHB). Each experiment was performed twice, independently. Molecules as well as their corresponding class, tested concentration and medium used are listed in Table 1. Each step of the protocol is described in detail in the Materials and Methods section.

**Table 1:** List of antibiotics, corresponding MoA, medium and concentration (as a factor of the MIC) used during incubations.

MoA (Functional class)	Sub_MoA	Chemical class	Chemical Subclass	Molecule	Concentration and medium
Cell wall synthesis	Bind to penicillin binding proteins and impair cell wall synthesis and integrity	β-lactam	Penicillin	Amoxicillin Ampicillin Piperacillin Mecillinam	1 × MIC-MHB 1 × MIC-MHB 1 × MIC-MHB 1 × MIC-MHB
			Carbapenem	Imipenem Meropenem^a^	1 × MIC-MHB 1 × MIC-MHB
			Cephalosporin	Cefazolin Ceftazidime	1 × MIC-MHB 1 × MIC-MHB
	Inhibit MurA	Organic Phosphonic Acid	–	Fosfomycin	1 × MIC-MHB
DNA synthesis	Inhibit DNA gyrase / topoisomerase IV	Quinolone	Fluoroquinolone	Ciprofloxacin^a^ Levofloxacin^a^ Moxifloxacin	1 × MIC-MHB 1 × MIC-MHB 1 × MIC-MHB
RNA synthesis	Inhibit RNA synthesis	Ansamycin	Rifamycin	Rifampicin Rifapentin	1 × MIC-MHB 1 × MIC-MHB
Protein synthesis	Bind to 30S rRNA and inhibit protein synthesis	Aminoglycoside	–	Kanamycin Tobramycin	2 × MIC-MHB 2 × MIC-MHB
		Tetracycline	–	Tetracycline Lymecycline	2 × MIC-MHB 2 × MIC-MHB
	Bind to 50S rRNA and inhibit protein synthesis	Amphenicol	–	Chloramphenicol Thiamphenicol	1 × MIC-MHB 1 × MIC-MHB
	Inhibit isoleucyl tRNA synthetase	Pseudomonic acid	–	Mupirocin	1 × MIC-MHB
Cell membrane	Targets LPS	Polypeptide	Polymyxin	Colistin	1 × MIC-CAMHB
Folate synthesis inhibition	Inhibit dihydrofolate reductase and dihydropteroate synthase	Anisole-Sulfonamide	–	Trimethoprim- Sulfamethoxazole^b^	8 × MIC-MHB

^a^For these molecules, the MIC-MHB may have been over-estimated (See Supplementary Table S1). ^b^Trimethoprim-Sulfamethoxazole were used as a mixture in the proportions 1:19 (Clinical and Laboratory Standards Institute, 2024b).

### Phase image dataset generation

**Figure 1:**
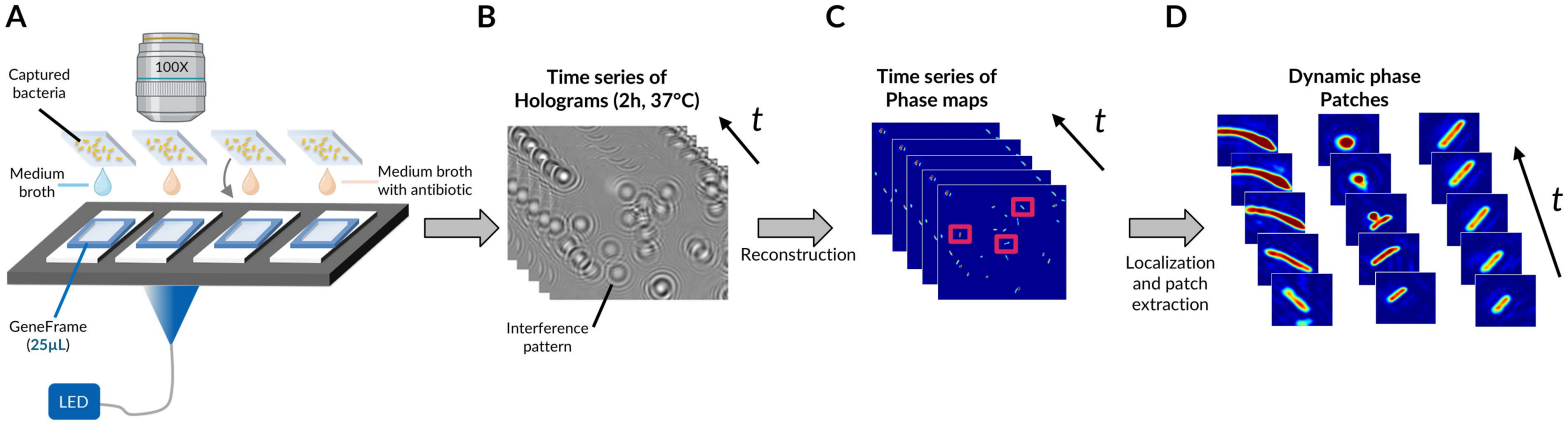
Pipeline for phase image database generation. **(A)** Time-lapse DIHM acquisition for up to 4 conditions (e.g., untreated and treated with 1 molecule at 3 different concentrations). **(B)** Recorded time-lapse holograms for one field of view. **(C)** Reconstructed time-lapse phase maps for the same field of view. **(D)** Dynamic phase map patches resulting from time frame registration and segmentation of bacteria. Microscope objective created with BioRender.com.

Figure 1 represents the overall analysis pipeline for the phase image database generation. In short, after an in-broth preculture aimed at bringing bacteria into a metabolically active state and allowing some replication, bacterial cells were immobilized via an electrostatic capture and placed in the medium and antibiotics (or no antibiotics in the case of untreated controls) in a transparent glass chamber suitable for time-lapse DIHM (Figure 1A; Supplementary Figure S1). Holograms were acquired every 3 min for 2 h (Figure 1B). Holograms are out-of-focus intensity images featuring interference patterns coding for phase information about the individual diffracting bacteria. Phase images were retrieved following a reconstruction of the acquired holograms, involving an optical-wave propagation algorithm (in the complex plane) based on the theory of diffraction of light. Each hologram was reconstructed to obtain in-focus phase images of each field of view and time-point (Figure 1C; Supplementary Figure S2). We localized the individual bacteria in each field of view and generated dynamic patches (simply referred to as “patches” in the rest of the manuscript), each one being a series of temporal images of a single bacterium (Figure 1D).

**Figure 2:**
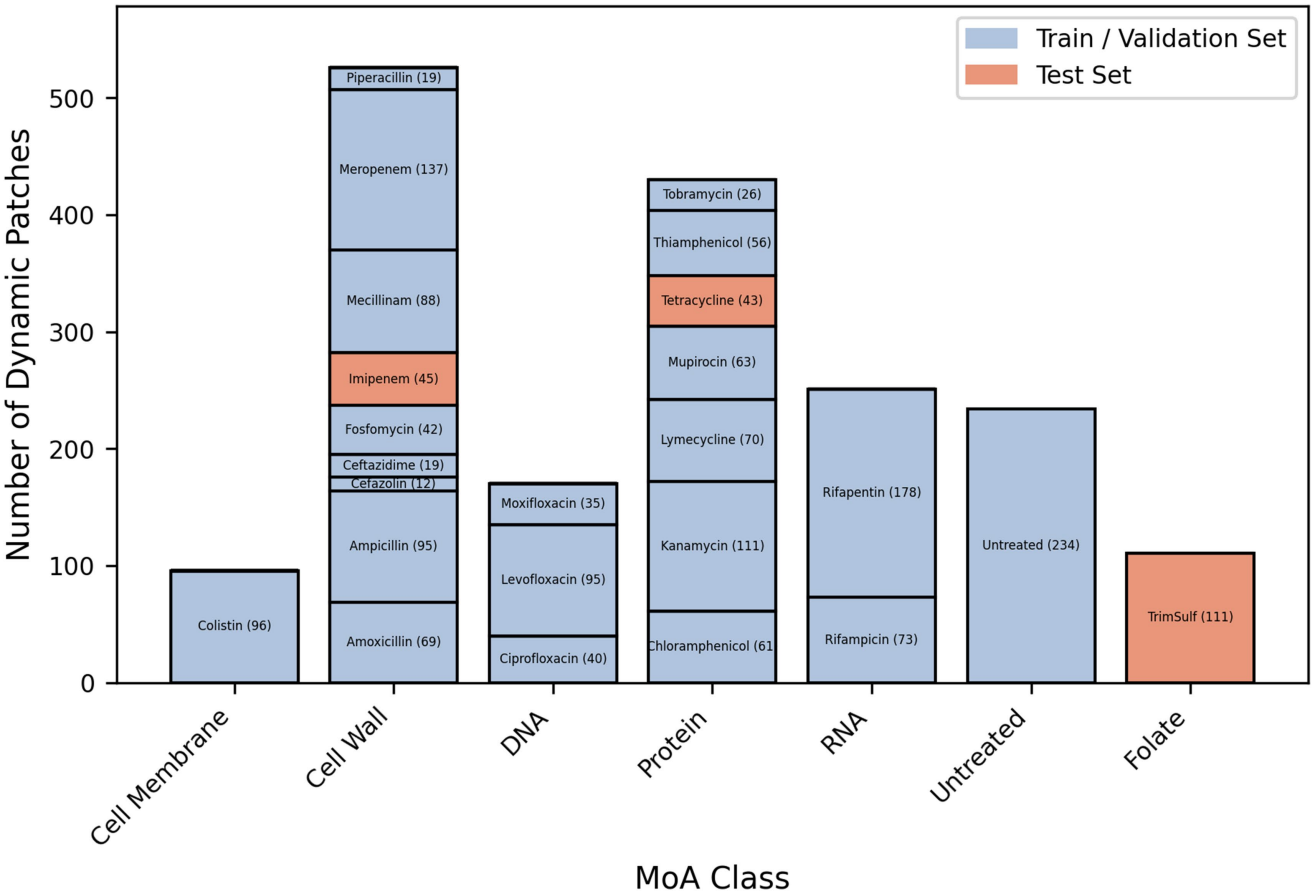
Number of single-bacterium dynamic patches per antibiotic (post preprocessing and cleaning) belonging to the 5 “conventional” MoA classes of this study (i.e., DNA, RNA, Protein, Cell Wall, Membrane) and to the “novel” MoA class (i.e., Folate synthesis inhibitor), as well as to the “untreated” control class.

**Figure 3:**
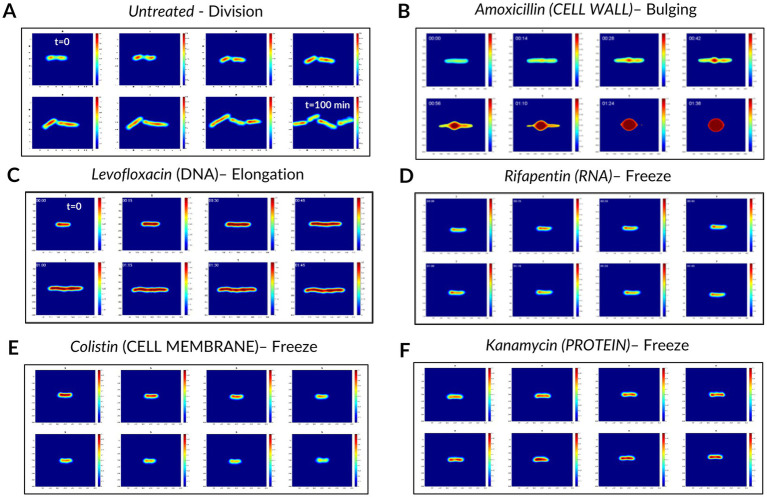
Examples of dynamic phase patches and phenotypes observed over 120 min of incubation without and with antibiotic treatments. **(A)** Division (untreated control). **(B)** Elongation and bulging with strong phase increase. **(C)** Elongation and global phase increase. (**D–F**) Frozen morphology and possibly evolving phase change. While images were acquired for 120 min every 3 min, here we show one image every 15 min. The phase scale (i.e., the colorbar) ranges from 0 to 0.5.

After having applied this pipeline to the molecules listed in Table 1, we obtained a dataset of single bacterial patches. These patches were preprocessed to remove the ones corresponding to a bacterium fully or partially detached from the coverslip (e.g., when a bacterium grew as long filaments), or to incomplete twin image artifact removal during the hologram reconstruction (see Materials and Methods), resulting in unusable images. The preprocessed final dataset was composed of 1818 single bacteria patches, each of them being constituted of 40 timeframes of 256 × 256 pixels vignettes. The patches split across the 5 conventional MoA classes, the novel MoA class, and the “untreated” bacteria class. The training and validation sets for the classification and novelty detection models contained patches from antibiotics of the conventional classes (except for 2 withheld molecules) and the untreated class. The novelty detection test set consisted of patches from the novel class and the 2 withheld conventional-class molecules (Figure 2), in order to provide both positive and negative controls. Examples of patches for each studied molecule are available in the supplementary material (Supplementary Figure S3). Some of the observed phenotypes over 2 h incubation are illustrated in Figure 3. Visually, the division phenotype of untreated bacteria (Figure 3A) can be distinguished from the absence of division in treated bacteria (Figures 3B–F). Among the treated bacteria, more specific phenotypes emerge depending on the class of drug. Molecules of the cell wall class induce strong morphological changes such as elongation, filamentation, swelling, bulging and/or lysis (i.e., beta-lactams), depending on the specific molecule and drug concentration (Supplementary Figure S3). These morphological changes correlated often with an increase of the phase (i.e., redder colors on the phase images, Figure 3B). Molecules of the DNA class tended to induce an elongation phenotype and increased phase (Figure 3C). Molecules of RNA, cell membrane and protein classes caused similar phenotypes, characterized by a fast freeze of the bacterial morphology; the phase evolution is not easily interpretable by eye for these 3 classes (Figures 3D–F).

### MoA classification

**Figure 4:**
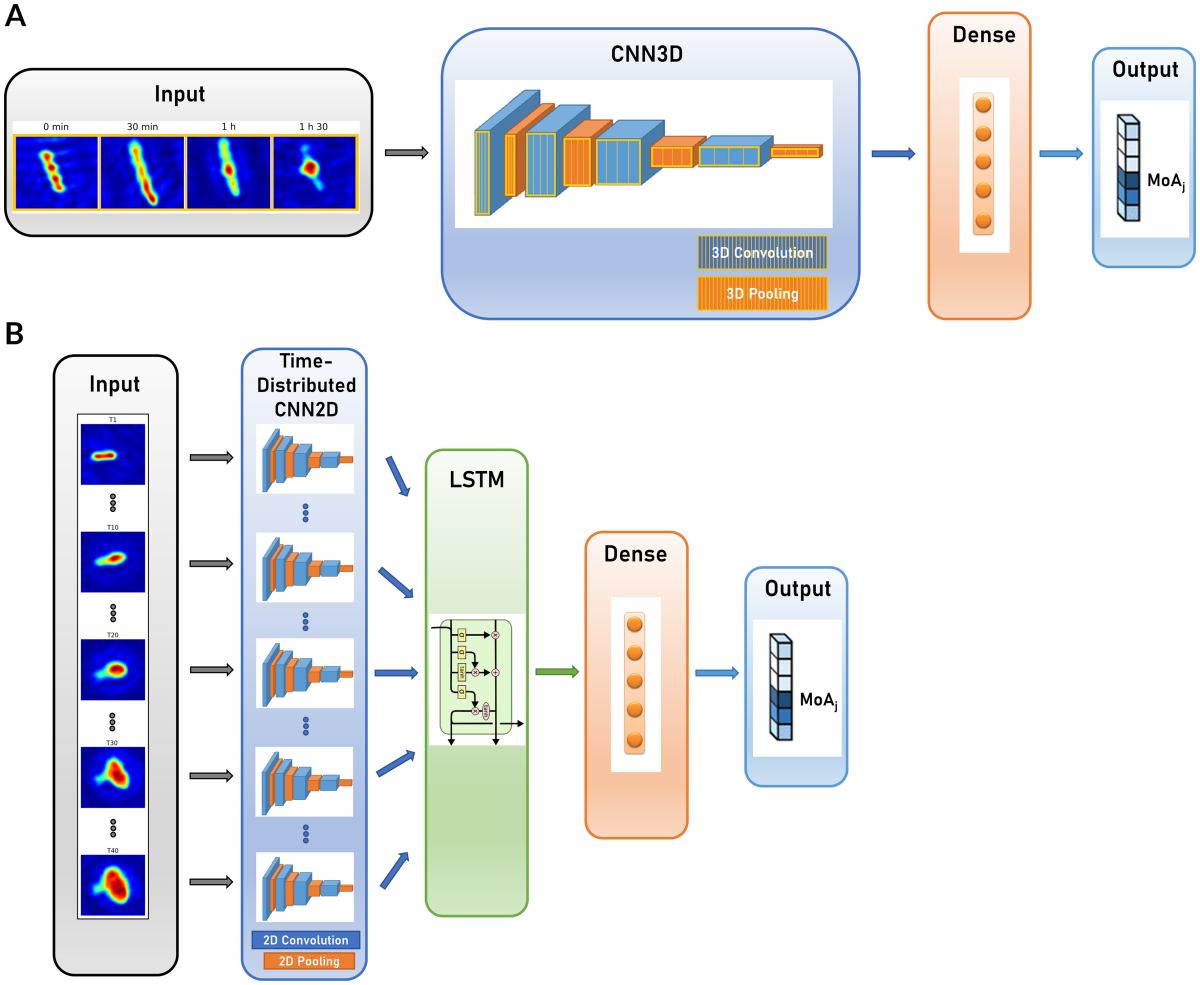
Compared CNN architectures for dynamic patch MoA prediction. **(A)** 3-dimensional Convolutional Neural Network (CNN3D); **(B)** Convolutional Recurrent Neural Network (CRNN).

We implemented DL classification models to predict the MoA associated with each patch among the 5 conventional classes and the untreated class. We considered 2 main models, described in Figure 4: CNN3D and CRNN. In CNN3D (Figure 4A), the time dimension was analyzed in the same manner as the X and Y space dimensions, using 3D convolutional layers, though with different kernel sizes between time and space. 3D convolutional layers were alternated with pooling layers; classification was achieved through final fully connected dense layers. In CRNN (Figure 4B), the time dimension was treated with a dedicated recurrent layer (Long-Short Term Memory – LSTM, or Gated Recurrent Units - GRU). This layer was incorporated on top of the time-distributed 2D convolutional and pooling layers, followed by the addition of fully connected layers.

**Figure 5:**
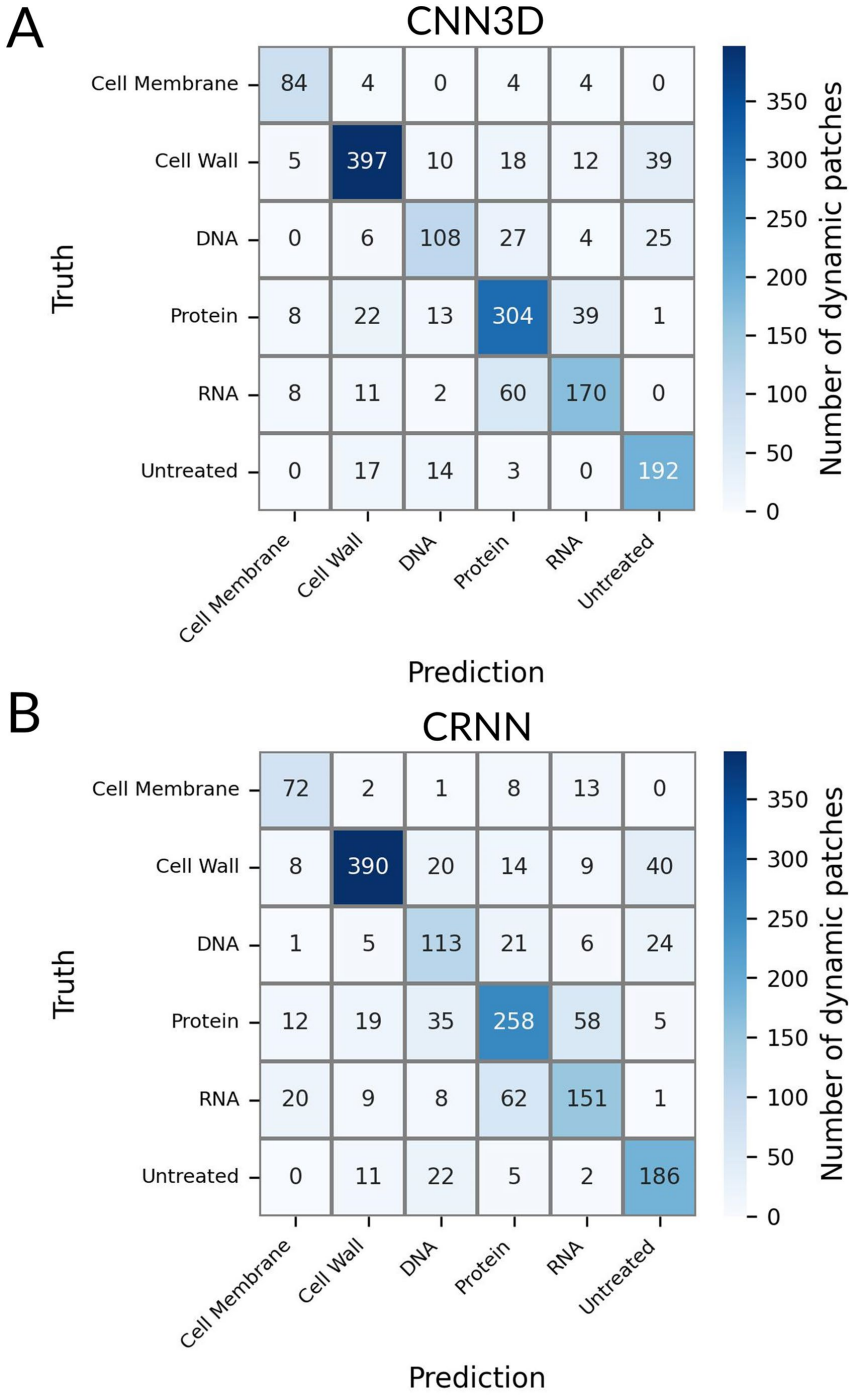
Confusion matrix associated to the Deep-learning-based classification for the untreated class and for 5 conventional MoA classes: Cell Wall synthesis inhibitors, Cell Membrane function inhibitors, Protein synthesis inhibitors, DNA synthesis inhibitors and RNA synthesis inhibitors. **(A)** Patch-level classification (CNN3D model) **(B)** Patch-level classification (CRNN model).

We used a fast genetic optimization algorithm to optimize the value of hyperparameters such as number of layers, number of units in each layer or kernel sizes (Deb et al., 2002). We computed the final performance of CNN3D and CRNN models using 10-fold stratified cross-validation. We focused on accuracy (i.e., overall percentage of correct predictions) and F1 score (i.e., the harmonic mean of precision and recall, used to study performance in the case of unbalanced classes). The best model for patch classification was CNN3D, for which we obtained an accuracy of 78%, versus 73% for CRNN, and a F1 score of 77% for CNN3D, versus 70% for CRNN. Both models’ confusion matrix and classification report are shown in Figure 5 and Supplementary Figure S4, respectively. The best performance was achieved for the cell wall class, with F1-scores of 85%, probably because these molecules induce very specific filamentation or bulging phenotypes (Figure 3A). The main misclassifications were observed between RNA and protein classes, for which the size and shape of the bacteria seemed frozen (Figures 3D,F).

In a second step, we studied the model performance at sample and antibiotic levels by aggregating individual patch predictions. Indeed, in a real-life application, the technology would be applied to assess the MoA from a sample or a collection of samples using the same antibiotic. Hence, to predict the MoA from one sample, we performed majority voting among all its patch predictions. To predict the MoA of one antibiotic, majority voting was applied to all patch predictions from all replicates associated to the considered antibiotic. This process resulted in a prediction of the correct MoA for 90% of the samples and 86% of the antibiotics for the CNN3D model, while the MoA of 95% of the samples and antibiotics were correctly predicted for the CRNN model (Table 2). Full classification results per sample and antibiotic are displayed for both models in Supplementary Table S2. Overall, model performance was greatly enhanced compared to patch-level, which highlights the discriminative power of our pipeline at the slide and sample levels despite inherent experimental and biological variability at single cell level.

**Table 2:** Classification accuracy at patch, sample and antibiotic levels for different Deep Learning models.

	Accuracy (%)	
	CNN3D	CRNN
Patch-level	78	73
Sample-level	90	95
Antibiotic-level	86	95

The lone misclassification of CRNN is observed for piperacillin which was classified as DNA while belonging to the cell wall class. CNN3D confused 3 molecules – moxifloxacin, piperacillin, ceftazidime – with the untreated class. As shown in Figure 2, only a small number of patches represented these molecules in the dataset, because their important filamentation phenotype resulted in the detachment of many bacteria from the coverslip, which made the hologram reconstruction and segmentation too challenging. This led us to include patches with rather weak filamentation effects, resembling bacteria from the DNA samples or about to divide (i.e., similar to the untreated bacteria). This made it more difficult for the model to learn how to classify them.

Interestingly, CRNN showed better performance at the macro level while being less accurate at the patch level, compared to CNN3D. This comparison at the macro level should not be considered as very significant: sample-level and antibiotic-level accuracy (resulting from an aggregation of patch-level predictions) are not robust metrics, given the limited number of antibiotics (i.e., 22) and samples available. Thus, two molecules (out of three) are misclassified by CNN3D because the number of correctly classified patches equals the number of patches that are confused with the untreated class. A slight improvement of the model, for example by adding more patches for these 2 molecules in the dataset would result in equally good macro level accuracy (i.e., ~95%) for both CNN3D and CRNN models.

### MoA novelty-detection

**Figure 6:**
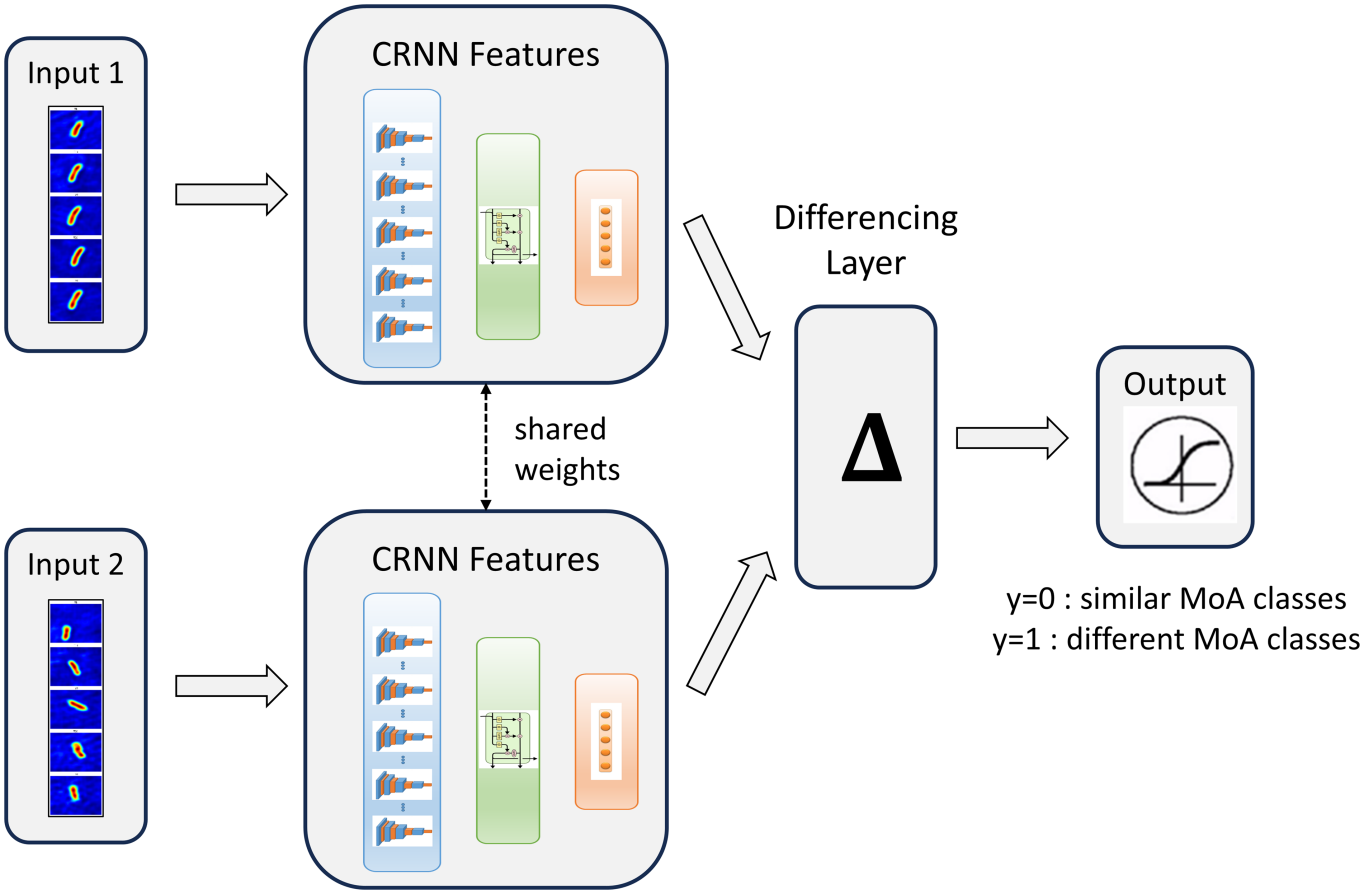
Siamese Convolutional Recurrent Neural Network (sCRNN) architecture for dynamic patches MoA similarity prediction. We call *y* the network prediction.

This work demonstrated the ability of our technology to discriminate known MoA classes using a standard supervised classification scheme. Still, the most needed application for drug discovery is the capacity to predict the novelty of the MoA of an antibiotic candidate compared to a set of known MoA classes. Thus, we proposed a novelty detection framework based on a Siamese Neural Network (SNN). This model was composed of 2 CNN3D (sCNN3D) or 2 CRNN (sCRNN) with shared weights, trained in parallel and combined with a differencing layer to predict whether 2 patches correspond to similar (y = 0) or different (y = 1) MoA classes (Figure 6). During the inference phase, patches from an unknown antibiotic candidate can be compared to the ones from known classes using the trained network. If the candidate exhibits a novel MoA, the network will predict most pairs as different. Our novelty assessment framework is illustrated in Supplementary Figure S5.

We designed an experiment to explore the potential of this approach. We defined as “known” dataset the patches from the 5 conventional MoA classes (except imipenem and tetracycline), and the untreated class. We considered as “unknown” imipenem and tetracycline, as well as trimethoprim-sulfamethoxazole (“trim-sulf”) which belongs to the class of folate biosynthesis inhibitors. Given that trim-sulf MoA class is different from the conventional MoAs in our training dataset, we expected trim-sulf to be predicted as having a novel MoA by our pipeline, while imipenem and tetracycline were expected to be predicted as having a known MoA. We trained our SNN on 70% of the patches from the known dataset. We kept 10% of patches in a validation set to compute the intrinsic SNN performance. The remaining 20% were included in a test set, along with patches from the unknown candidates that would be used in the inference phase to compute the novelty scores. We generated all pairwise combinations of patches from the training and validation sets to train and evaluate the SNN. We obtained 850,860 training pairs and 10,440 validation pairs.

We trained both sCNN3D and sCRNN models to compare their performances. Global performances were similar, but we decided to keep sCRNN for the rest of the experiment as it offered better generalization for the small classes and its training time was twice as fast compared to CNN3D. sCRNN achieved an accuracy of 86% and an F1-score of 76%. Full performance metrics and confusion matrix are shown in Supplementary Figure S6. The model showed good performance at detecting pairs of patches with different MoAs, as the recall for the class y = 0 was 92%. On the other hand, the model frequently misclassified pairs with same MoA, as the recall for the class y = 1 was 51%. This result is understandable given the high biological and experimental variability we observed at the bacteria level.

**Figure 7:**
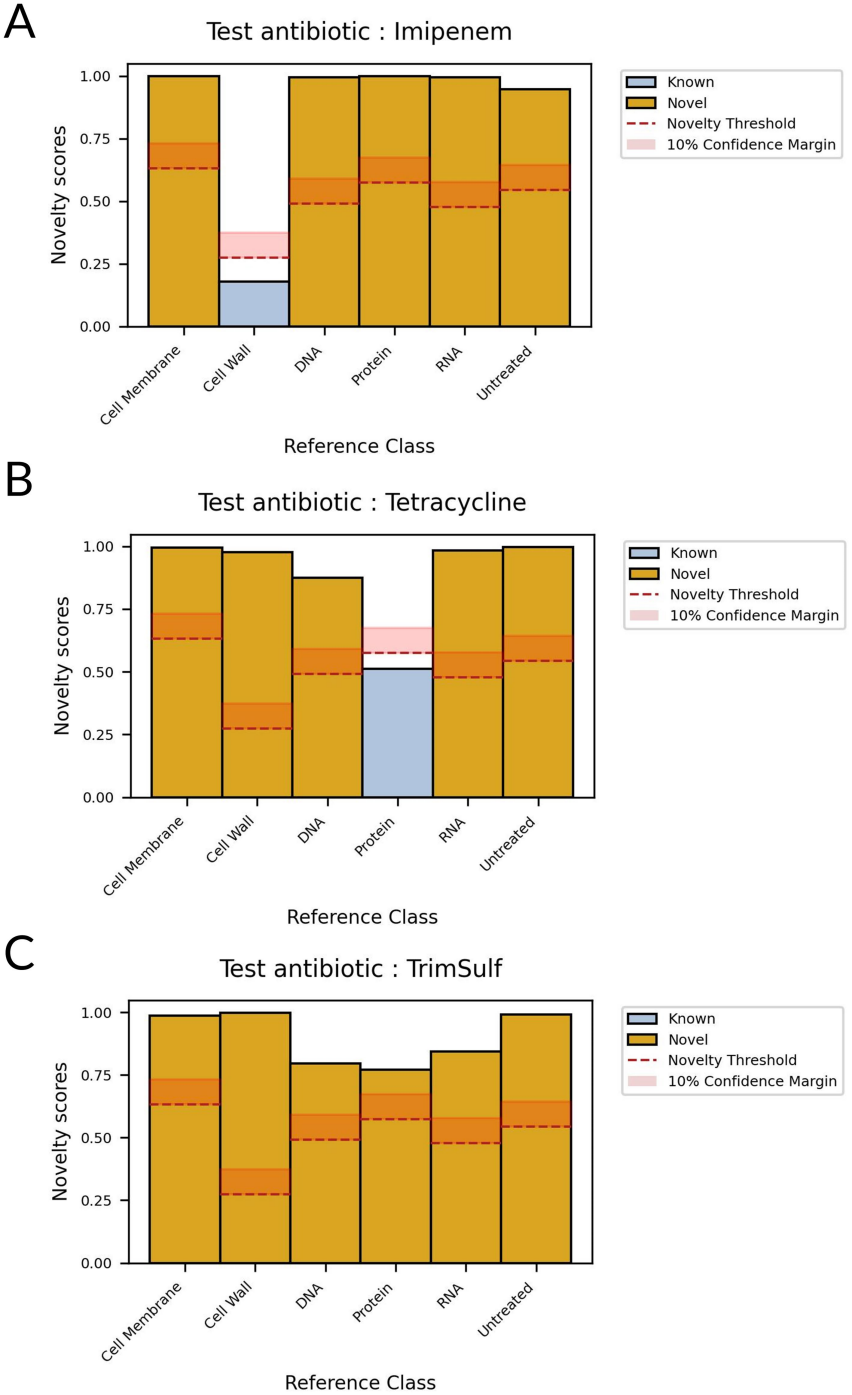
Novelty scores for different test antibiotics with respect to known classes. If all scores are superior to novelty thresholds, the test antibiotic is detected as novel. **(A)** Test antibiotic: imipenem **(B)** Test antibiotic: tetracycline **(C)** Test antibiotic: trim-sulf.

Then, during the inference phase, we compared all patches from the test set to the ones in the known set. We aggregated sCRNN predictions for each known class and unknown candidates to compute novelty scores, shown in Supplementary Figure S7. The novelty scores range between 0 and 1, 0 meaning that the test candidate exhibits a MoA very similar to the training class, 1 meaning that it is very different. We defined a novelty threshold for each known class that corresponds to the novelty score of the class when comparing its training and test data. The novelty threshold is expected to assume a mid-to-low value, depending on the intrinsic variability of the class and the ability of the model to generalize it. If the novelty scores of an unknown candidate are superior, with a significant confidence margin, to the novelty thresholds of all known classes, the unknown class can be considered as novel. We observed that our experiment validated the proposed framework (Figure 7): trim-sulf MoA (i.e., folate synthesis inhibitor) was predicted as novel since it was assigned novelty scores significantly higher than the corresponding novelty thresholds for all other MoAs. On the other hand, the two withheld molecules from the conventional classes, imipenem and tetracycline, exhibited small novelty scores with respect to their corresponding classes only, i.e., cell wall and protein, respectively.

## Discussion

Herein we have developed a fast, safe and label-free technology to classify antibiotic MoA among 5 functional classes and detect the MoA novelty of an additional antibiotic class using *E. coli* ATCC 25922. This technology combines DIHM and DL. It has proved promising for the identification of antibiotics with known MoA (i.e., whose MoA is in the database) and for detecting the MoA novelty of new antimicrobial candidates. Importantly, it considers the effect of time on antibiotic action (time-lapse data acquisition). In a context where the determination of a drug MoA remains difficult but important to facilitate preclinical progression, the proposed technology would be more advantageous than the classically used MMS technique.

### Time-lapse DIHM reveals early morphological and phase changes during antibiotic treatment

Time lapse image acquisition allows a focus on rapid antimicrobial effects on the morphology and phase of single bacterial cells, enabling users to elucidate the antimicrobial MoA in less than 2 h. Changes of bacterial area or mean-phase-intensity can be seen as early as 30 min, notably for the DNA synthesis inhibitors (Supplementary Figures S3, S8, S9). Bacterial morphological changes following antimicrobial treatment (Cylke et al., 2022) have already been observed in phase contrast (Zahir et al., 2019), electron (Cushnie et al., 2016) and fluorescence (Nonejuie et al., 2013; Salgado et al., 2025) microscopies. The resolution and contrast obtained with the simple and cost-effective DIHM configuration combined with appropriate hologram reconstruction is similar to the more expensive and bulkier optical sectioning microscopy involved for instance in the original BCP technology (Nonejuie et al., 2013). Both techniques show changes in the shape of the cells. The information retrieved specifically by holographic microscopy is quantitative phase and its 2D heterogeneity within the bacterium area, while fluorescence images reveal stain-specific parts of the bacterium, typically its cell membrane and DNA. We can hypothesize that DL models base their classification on both morphology and phase information (Supplementary Figure S10). It is interesting to note similarities between phase nodes observed with DIHM and DNA staining observed via fluorescence microscopy, as illustrated in Supplementary Figure S11. When compared to phase contrast microscopy, DIHM enables the visualization of high phase nodes before the actual outer morphology changes, for instance in the beta-lactam-induced bulging phenotype as illustrated in Supplementary Figure S12. Moreover, DIHM does not require precision focusing mechanics as holograms are deliberately acquired out of focus; it is also insensitive to autofluorescence of the sample unlike fluorescence microscopies.

The imaging technology could be coupled with microfluidic approaches for parallelizing many more conditions, for automated handling of antibiotic dilutions and delivery to the incubation chambers, and to start imaging from t_0_ of contact of the bacteria with the tested molecules (to avoid the currently blind first 15 min). Moreover, the electrostatic capture of the bacteria aiming at their immobilization on the glass substrate to facilitate time-lapse imaging could be replaced by a physical constraint in capillaries on specific microfluidic devices such as a “mother machines” (Allard et al., 2022), which would prevent bacteria from moving out of the plane when growing or filamenting; this would help to increase the number of usable reconstructed phase images. In addition, DIHM system can be cost-effectively duplicated (See the list of elements in the Materials and Methods section and Supplementary Figure S1, with motorized stages and the objective as the more costly items) and would be easily adapted to high throughput screening systems, in upright or inverted configurations. Moreover, the required quantity of the tested molecule is minimal (i.e., 25 μL of solution during the treatment phase), which is of importance at the early drug development steps, when available quantities of compounds can be limited.

### DL model performance

Our final DL model detected the MoA of known antibiotics with high performance (i.e., 95% macro level accuracy). Our fully automated analysis pipeline treats single-cell images containing both phase and time information (i.e., 256 pix × 256 pix × 40 time-points per single-cell). The information potential of such high dimensionality data would require more examples to be maximized. The risk with a too-small dataset is the lack of generalization. Indeed, the weak signals are more likely to be ignored due to the lack of examples. It also constrains the models to be carefully tuned to avoid overfitting. In the classification work presented herein, the performance is limited for the Cell Membrane class represented only by colistin, as well as for some molecules of the Cell Wall class (i.e., piperacillin and ceftazidime) that are represented by a low number of bacterial cells compared to the other molecules. For these two beta-lactams, the poor classification performance is worsened by the fact that the patches passing the data curation criteria are typically the ones showing small filamentation phenotype (by opposition to very long filamentation), therefore resembling the division phenotype of the untreated class — indeed, filamentous cells detaching partially or fully from the substrate over the course of the incubation are eliminated during the automated cleaning of the image dataset. Moreover, the significant cell-to-cell variability cannot be fully avoided, and AI tools may be even more sensitive to it when dealing with time-lapse data. Increasing the size and representativity of the image dataset would improve the classification performance.

The highest misclassification rate at single bacterium level was observed between RNA and protein synthesis inhibitors (Figure 5), both showing almost no morphological changes after treatment (Figure 3; Supplementary Figure S3). This may be explained by the fact that protein synthesis, downstream of RNA synthesis, could be blocked as a side effect of RNA synthesis inhibition, which could lead to closely related holographic phenotypes. It is possible the risk of misclassification rate could be reduced by using different antibiotic concentrations or treatment times. Indeed, previous MMS assays testing several antibiotics have shown that these parameters have an impact on the detection of on-target effects, but also of secondary effects or off-target effects (Cunningham et al., 2013). Nevertheless, we were able to discriminate both of these MoAs at the molecule level (Tables 2; Supplementary Table S2). Visual inspection of examples of patches (Supplementary Figure S3) suggests that subtle differences between molecules from the same class can be distinguished. This is visible in the cell wall class where, for instance, imipenem and meropenem (i.e., carbapenems) generated swelling of the bacteria while ceftazidime (i.e., a cephalosporin) induced filamentation (Supplementary Figure S3), even though both are beta-lactams. This may be explained by the different penicillin binding proteins targeted by each beta-lactam, as reviewed previously (Cylke et al., 2022). These observations show the power of holographic microscopy and suggest that the proposed automated method to detect MoA novelty could also be used to detect even more subtle differences between antimicrobial candidates.

### Focus on the novelty detection feature

One of the main steps forward of HoloMoA compared to the state-of-the-art lies in the proposal, for the first time to our knowledge, of an automated DL-based pipeline for MoA novelty detection, which could ease the detection of new valuable antimicrobial candidates. We evaluated our novelty detector on a test set containing three unseen molecules (i.e., absent from the training dataset): imipenem and tetracycline belonging to the conventional functional classes (represented by other molecules in the training set) and serving as negative controls regarding novelty detection, and trimethoprim-sulfamethoxazole representing a totally unseen class and serving as positive control regarding novelty detection. Our novelty detector successfully identified the conventional MoAs of imipenem and tetracycline while detecting the “novelty” of trimethoprim-sulfamethoxazole. Our approach for MoA-novelty detection requires relatively high computing resources for siamese neural network training and inference. This is due to the large combinatory arising from the building of pairs to feed the network, associated with the high intrinsic dimensionality of our data. This computing cost prevents us from further optimizing the hyper-parameters of the networks. Some solutions can be explored to mitigate this computing cost, such as applying class-specific subsampling rates or applying a time–space subsampling of patches themselves. Otherwise, lighter DL architectures could be explored for novelty detection such as the use of convolutional auto-encoders (Hasan et al., 2016); they would reconstruct data from novel antibiotic candidates with a higher loss than the data from known antibiotics on which the auto-encoder would have been trained. Moreover, we observed that the MoA class with the lowest statistics (i.e., cell membrane) gets the highest novelty threshold (Figures 2, 7), due to the lack of generalization of the siamese network. We expect that increasing the size and representativity of the image dataset will improve the reliability of our AI tools.

### The use of a temporal dimension

We made the choice in our experimental design to include a longitudinal dimension in the data to capture dynamic patterns over time. Though our approach raises challenges, such as the high computational cost of the model training and the need of large number of examples to leverage the high complexity of the data, we verified that the inclusion of 40 time points offers better MoA classification performance compared to using fewer time points, or only an end point. Indeed, we reoptimized and retrained classification models using 5 time points (every 30 min) and one end point (after 1 h) and the accuracy decreased by 8 and 23%, respectively, at the patch level (Supplementary Figures S13, S14). Performance was also worse at the sample and antibiotic levels (Supplementary Table S4) when decreasing the number of time points. Hence, time dimension can be an asset for MoA characterization studies if treated with an appropriate algorithm, though the number of time points should be selected carefully to balance complexity and accuracy.

### Choice of concentration

Originally, it was intended to use 1 × MIC concentration of antibiotics to allow comparison with broth microdilution antimicrobial susceptibility testing. However, this was not possible in all cases in the 2 h-long HoloMoA format. We based our choice primarily on visual inspection of the time-lapse images, identifying the concentration at which the phenotypic response clearly diverged from the untreated samples. To increase throughput and reduce bias, it would be desirable to automate this step in the future. For example, one could compute novelty scores for each concentration of an antibiotic with respect to an untreated class and select the lowest concentration displaying a significant score. As an illustration, we applied the novelty detector (see description in 2.4) to trim-sulf used at 0, 1, 2, 4 and 8 × MIC (Supplementary Figure S15). We observed that all concentrations below 8 × MIC were assigned a novelty score with respect to the untreated class similar to the novelty threshold of this class, whereas the 8 × MIC sample got a 0.99 novelty score. This result validates the choice of 8 × MIC as the selected concentration for our trim-sulf experiment.

### Interpretability

One limitation of our study lies in the interpretability of the deep learning models used for classification and novelty detection. For example, our models showed good ability to differentiate cell membrane class from RNA and protein classes, while they present similar visible phenotype (ie freezing of the bacteria), but it remains difficult to determine which specific features the models relied on for such discrimination. The internal decision-making process of our models is difficult to interpret due to their intrinsic architecture, involving a large number of parameters and hierarchical feature representations. Although interpretability techniques such as saliency maps can offer insights for models such as CNN2D, their extension to CNN3D or recurrent networks remains less straightforward, computationally intensive and is an active area of research. Future work may focus on integrating explainability methods tailored to these architectures to better understand the biological or morphological features that differentiate the classes.

### Summary on improvements and perspective

Among technological improvements foreseen, we will explore improved immobilization (in the plane) and increased throughput to increase the statistics of image data feeding the AI. We will also test the simplification of the image analysis pipeline, optimizing the number of time-points, full-field images rather than single-bacteria patches, and even go much further and not reconstruct at all the holograms (i.e., use them as is). While bacterial synchronization is not trivial, reinforcing the bacterial preculture step with successive in-broth cultures (Cutler and Evans, 1966) could reduce the cell-to-cell heterogeneity. Regarding the biological model, the next step would be to increase the number of molecules in the dataset to reinforce the demonstration by feeding the tool with more phenotype examples, more variability, more off or dual-target cases, etc. The novelty detector should also be tested on novel antimicrobial candidate molecules. In the longer term, the technology could be implemented for other pathogens including the ESKAPE pathogens, but also fungi and parasites. Finally, vibrational spectroscopy such as Raman spectroscopy is an appealing label-free modality to use in combination with DIHM. Indeed, Raman micro-spectroscopy would provide the complex chemical fingerprint of the treated bacteria (Athamneh et al., 2014), which would complement the morphological and phase information rendered by the DIHM modality and help MoA prediction.

To conclude, thanks to a combination of DIHM and DL methods, HoloMoA demonstrated promising performance for antimicrobial MoA classification and novelty detection, which remain important but challenging requirements during antimicrobial discovery and development. We believe that this tool can support antimicrobial drug developers and, thus, contribute to improving the antimicrobial pipeline and tackle the ongoing AMR pandemic.

## Materials and methods

### Strains, media, and antibiotics

*E. coli* ATCC 25922 was purchased from LGC. Antibiotic powders were dissolved in the appropriate solvent according to manufacturers’ instructions and the resulting stocks were aliquoted and stored at −20°C for a maximum of 6 months. Each aliquot was used only once. MIC were determined following CLSI procedures for broth microdilution antimicrobial susceptibility testing (Clinical and Laboratory Standards Institute, 2024a). Both MHB (70,192, Sigma) and CAMHB (212,322, BD) were used: MIC values obtained in CAMHB were compared to the CLSI performance standards (Clinical and Laboratory Standards Institute, 2024b) to validate the antibiotic stock solutions. MIC values obtained in MHB were used to design experiments for holographic microscopy analyses, except for colistin which was handled in CAMHB only. The antibiotic MICs are listed in Supplementary Table S1.

*Preparation of the bacteria for holographic microscopy analyses* was carried out as follows. Bacteria from glycerol stocks were thawed and sub-cultured on Columbia agar with 5% sheep blood (COS) (43,041, bioMérieux) and incubated overnight at 37°C. The obtained plate was kept at 4°C for a maximum of 3 weeks. Before any further experiment, a single isolated colony was streaked on a new COS plate and incubated overnight at 37°C. The next day, 10 mL MHB or CAMHB were inoculated with isolated colonies to the 0.5 Mc Farland Standard (McF) (Densimat, bioMérieux) and incubated at 37°C, with shaking at 250 rpm for 2 h. Post liquid preculture, 2 × 1 mL were centrifuged for 5 min at 2000 x g at room temperature. After removal of the supernatant, pellets were resuspended in 1 mL of 20 mM phosphate buffer (Phosphate buffer 20 mM; NaCl 50 mM; pH 7.2) and centrifuged for 5 min at 2000 x g at room temperature. Pellets were resuspended and pooled in 1 mL phosphate buffer. Turbidity was adjusted to 0.5 McF with phosphate buffer. 120 μL of the suspension were deposited on a 170 μm thick aminosilane-coated glass coverslip (1,666,121, Schott Nexterion) and allowed to sediment for 15 min at room temperature to allow electrostatic capture of the individual bacterial cells on the coverslip. The coverslip was washed 3 times with the phosphate buffer and once with MHB either with or without antibiotic, while taking care the surface never dried. The coverslip was held with the capture side facing down and placed in a 25 μL incubation chamber, so that the captured bacteria remained in contact with the medium +/− antibiotic (Supplementary Figure S1B). The chamber was sealed with 10 × 10 × 0.25 mm^3^ of Gene Frame adhesive (AB0576, ThermoFisher Scientific) fixed on a standard glass microscope slide. The number of captured bacteria over the 10 × 10 mm^2^ surface area was approximately 1 × 10^5^ cfu, meaning an inoculum of ~ 10^6^ cfu/mL during incubation with the antibiotics in the 25 μL chamber; this inoculum is comparable to the one used during standard MIC measurement by broth micro-dilution (Clinical and Laboratory Standards Institute, 2024a). Up to four chambers and incubation conditions were prepared from the same culture and analyzed simultaneously.

*Time-lapse holographic microscopy* was carried out as previously described (Degoût-Charmette et al., 2025), with selected modifications. We used a purpose-built prototype of a digital inline holographic microscope (Supplementary Figure S1A). The light source was a fiber-coupled (M35L01 fiber, Thorlabs), high-power LED emitting in the blue wavelength (FCS-0455-000, Mightex). A 45° protected aluminum turning mirror (CCM1-G01, Thorlabs) enabled direction of the light vertically inside the simplified upright microscope. Light probed the sample held in a 4-slide holder (MLS203P10, Thorlabs); a XY motorized translation stage (NRT100 and MTS25/M-Z8, Thorlabs) enabled horizontal displacement of the samples. A coverslip-corrected 100X objective with a numerical aperture of 0.95 (PLFLN100X, Olympus) collected the transmitted and forward-scattered light; it was mounted on a Z motorized stage to adjust focus (MTS25/M-Z8, Thorlabs). The collected light was filtered by a bandpass filter centered at 450 nm with 10 nm full width at half maximum (FBH450-10, Thorlabs). An achromatic lens with 150 mm focal length (LA1433, Thorlabs) acted as tube lens and focused the collected light on a back-illuminated CMOS camera featuring 3,088 × 2076 pixels and 2.4 μm pixel size (UI-3884LE-M-GL, IDS). The effective magnification was ~86X. The entire holographic microscope fitted within a standard microbiology incubator set to 37°C. Time-lapse holographic imaging was performed for 2 h in an automated manner via a purpose-made Labview interface, at a rate of one hologram every 3 min and 8 fields of view per sample. For each field of view and time-point (tp), the routine consisted of acquiring one hologram when the sample was defocused by Δz = 20 ± 2 μm (Supplementary Figure S1C), and one background image when the sample was defocused by Δz = 200 μm. Each field of view represented 85 × 57 μm^2^ region of the coverslip and enabled the monitoring of ~10 captured bacteria. Calibration of the coverslip tilt caused a 15 min delay between the acquisition of the first image of the kinetics and the first contact of the bacteria with the antibiotic. In what follows, *t* = 0 refers to the start of the imaging.

### Hologram reconstruction

The hologram reconstruction algorithm (purpose-built, in Matlab) was mainly performed as before (Degoût-Charmette et al., 2025). In short, as a preliminary step, each acquired hologram was divided by its corresponding background to normalize and correct for invariant patterns linked to parasitic objects on the light path (i.e., typically dust on optical elements). Second, the reconstruction of the phase shift image (corresponding to the object-plane) from each hologram (corresponding to the image-plane) involved 2 main steps schematized in Supplementary Figure S2: digital re-focalization and twin-image removal. As illustrated in Supplementary Figure S2A, the propagation algorithm based on Rayleigh Sommerfeld diffraction theory (Goodman, 2005) enabled reconstructing a digital z-stack from each hologram; the refocused phase map corresponding to the object-plane was retrieved from the z-stack according to a maximum contrast criterium for almost pure phase objects (i.e., low absorbers) (Dubois et al., 2006). The twin-image artifact is intrinsic to the inline configuration of DIHM and disturbs the background of the reconstructed phase map and its subsequent analysis. We implemented a twin-image removal algorithm inspired by the Gerchberg-Saxton algorithm (Gerchberg, 1972) and its numerous variations, in particular Latychevskaia and Fink (2009). As illustrated in Supplementary Figure S2B, the algorithm consists in a series of alternating back and forward propagations between the hologram-plane and the object-plane, while imposing constraints on the propagating field in each of the two planes. In the hologram-plane, the squared modulus of the propagated field must be equal to the recorded hologram; in the object-plane, absorption must be positive while the phase shift is forced to 0 when it is lower than a certain adaptive threshold. Iteration after iteration, the reconstructed phase is updated and converges toward its exact value (corresponding to a null threshold).

### Dynamic patch generation

Following the reconstruction of the phase shift maps for each field of view and time-point, individual bacteria were localized via an image binarization to obtain dynamic patches (256 pix × 256 pix × 40 tp) centered on each individual bacterium. Given the strong movements and morphological changes of bacteria (i.e., while dividing or under certain antibiotic treatments), we performed the single-bacteria localization and patch generation on the average image of the 40 time-points. Before entering the machine learning pipeline, a rule-based preprocessing was applied to filter and clean the dynamic patches (simply referred to as “patches” in the rest of the section). The rules were derived from experts’ knowledge and exploratory statistics.

First, patches failing the quality checks were removed. Three criteria were applied to establish the quality of the images. Phase signal ranged between 0 and 0.5. Patches starting with pure background images were removed as they were due to segmentation issues. An image was considered as background if it fulfilled one of the following criteria: (i) the maximum phase was below a threshold of 0.3, (ii) the maximum phase was found in the edge of the image and the mean phase was below 0.02, where the edge corresponds to a band of 10 pixels width. Patches with background images at the end of the series were removed. This would correspond to a loss of the bacterium (typically a detachment from the glass coverslip). There was one exception: it could correspond to the lysis of the bacteria, and in that case, we kept the patch because lysis is an actual signature of the MoA of the antimicrobial. In case of lysis, a small amount of bacteria material remains (“footprint” of the bacteria), therefore we considered as lysis a series of background images at the end of the series with a mean intensity above 0.08. Patches with too many empty images were removed. Empty images were due to incorrect holographic reconstruction. These could be corrected with interpolation, as described in the next paragraph. However, we decided to limit the correction applied to the images, to preserve the original biological signal. Then, we removed patches that fulfilled at least one of these criteria: (i) the patches contained more than 10 empty images in total, (ii) the first image was empty, (iii) more than 2 images at the end of the series were empty.

Second, patches were cleaned by imputing some empty or background images. (i) Empty images at the end of a series were replaced by a copy of the last non-empty image. (ii) Empty or background images in the middle of the series were replaced by a weighted interpolation of the images before and after them:


(1) $$I_{i}=\frac{\left\{b-i\right\}}{\left\{b-a\right\}}I_{a}+\frac{\left\{i-a\right\}}{\left\{b-a\right\}}I_{b}$$

where I_i_ is the interpolated pixel values of the empty / background image, I_a_ and I_b_ the values of the first non-empty / bacteria images before and after it, i,a,b the corresponding indices inside the time-series (Equation 1). Interpolation of background images were only performed if only one background image was present between two bacteria images, as it could correspond to a rare, temporary, loss of track of the bacteria.

### Deep-learning-based MoA classification

Two DL-based models were developed for MoA classification. CNN3D (Figure 4A) consisted of a set of convolutional layers associated with maximum pooling layers, a set of fully connected layers combined with dropout layers and finally a classification layer. The convolutional kernels were 3-dimensional and compute convolutions along space and time dimensions. The kernel size was different between space and time dimensions. Convolutional layers activation function was Rectified Linear Unit (“ReLU”). We performed maximum pooling factor after each convolutional layer, except for the last one, after which we performed a global maximum pooling operation was performed to combine features and reduce their dimension. Activation functions of fully connected layers were ReLU, except for the classification layer which was a fully connected layer with softmax activation and a number of units corresponding to the number of MoA classes. CRNN (Figure 4B) consisted of a set of convolutional layers and maximum pooling layers which were time-distributed, a recurrent layer, a set of fully connected layers combined with dropout layers and finally a classification layer. Convolutional kernels were 2-dimensional and squared, activation functions were ReLU, maximum pooling and global maximum pooling were performed after the convolutional layers. The 2D convolutional networks were time-distributed, meaning that a network was applied to each image in the time-series, but shared the same kernels. Recurrent layers were either Long-Short Term Memory (LSTM) layers or Gated Recurrent Units (GRU) layers. The hyperparameters of the recurrent layers were not optimized compared to the original tensorflow implementation. Fully connected, dropout and classifications layers were defined in the same manner as for CNN3D.

Models were trained with Adam optimizer. The model’s loss was categorical cross-entropy. A maximum of 200 epochs was used for training, early stopping was applied with a patience of 15 epochs. Early stopping criteria was validation accuracy. The batch size was 8. Final model performance was assessed using a stratified 10-fold cross validation scheme. We optimized the value of various hyperparameters for both models: number of convolutional layers, number of kernels, kernel sizes, pooling sizes, number of fully connected layers, number of units per fully connected layers, recurrent layer type, number of units in recurrent layers, dropout rate, learning rate, class weight (meaning whether to re-weight inputs by a factor inversely proportional to the class size). The full list and the ranges that we considered for each model are presented in Supplementary Table S3. The last column contains the best value obtained after optimization which was picked for the rest of the workflow. The algorithm we chose for the optimization was the well-known, fast and elitist multi-objective genetic algorithm (Deb et al., 2002) “Nondominated Sorting Genetic Algorithm II” (NSGA-II) from Optuna library (Akiba et al., 2019). Optimization was done using 100 trials and using the model’s loss as metric. Contrary to the final model evaluation, the performance was computed using 1 validation fold to spare time. For the same reason, we only used a maximum of 100 epochs with early stopping and a patience of 10 epochs, reducing the batch size to 4.

To compute the classifier prediction at sample and antibiotic levels, we gathered all predictions, corresponding to all patches, from the 10 validation folds. We aggregated them at the desired level, and we performed a majority vote. We compared the resulting prediction to the true class to compute the model performance metrics at this level. If the majority vote resulted in equality between 2 classes, including the true class, we considered the prediction as wrong. The metric obtained for patches whose class corresponded to the majority class was called “patch performance” and could be interpreted as a confidence score associated to the sample or antibiotic class prediction.

### Deep-learning-based MoA novelty detection

Novelty detection was based on a siamese neural network (Figure 6) trained on a set of patches from known MoA classes. The siamese network consisted of two sub-networks sharing the same weights, a differentiation layer and an output layer. The sub-networks were two CNN3D or two CRNN, as described in the previous section with the only difference being that their final classification layer was removed. Their hyperparameters values were the same as the ones obtained after optimization for the classification approach. Each sub-network took as input a patch. The differentiation layer computed the L1 distance between the outputs of both sub-networks. Finally, the siamese output layer was a fully connected layer of two neurons with sigmoid activation, computing a prediction, y, which should be equal to 1 if the two input patches belong to different MoA classes and to 0 if they belong to the same MoA class.

Models were trained with Adam optimizer. The model’s loss was binary cross-entropy. A maximum of 10 epochs was used for training, early stopping was applied with a patience of 3 epochs. The batch size was 4. Model performance was assessed using a stratified validation set containing 10% of input data (135 patches). The training dataset consisted of 70% of patches from known classes, i.e., Cell Membrane, Cell Wall, DNA, Protein, RNA and untreated, corresponding to a total of 1,217 patches. As explained above, the input of the siamese networks was a pair of patches. To obtain the training pairs set, we computed all possible pairs, leading to a total of 850,860 pairs (676,565 corresponding to pairs of different MoA, i.e., y = 0, and 174,295 corresponding to similar MoA, i.e., y = 1). We got 10,440 validation pairs (8,358 corresponding to pairs of different MoA, i.e., y = 1, and 2082 corresponding to similar MoA, i.e., y = 0).

The trained siamese network was then used in inference mode for novelty detection. We computed all possible pairs between patches from the known dataset and the test dataset. The known dataset corresponded to the siamese network training and validation datasets, resulting in 1450 patches. The test dataset corresponded to the unknown MoA candidates (imipenem, tetracycline, TrimSulf) and a 20% subset of known classes, resulting in a total of 360 patches. Therefore, in total we inferred predictions with the siamese network for 522,000 pairs. These predictions were aggregated per pair of MoA classes or antibiotics between the known and the test dataset. For each combination, we computed a novelty score, corresponding to the mean of predictions according to the formula:


(2) $$S_{\mathrm{AT}}=\sum_{a,t}^{A,T}\frac{y_{\mathrm{at}}}{N_{A}N_{T}}$$

where y_at_ is the network prediction for a pair of patches from reference class A and test class or antibiotic T, N_A_ and N_T_ are the number of patches from reference class A and test class or antibiotic T (Equation 2).

To assess whether an unknown antibiotic corresponded to a novel MoA, the following rule was applied:


(3) $$\text{if}\;S_{\mathrm{AX}}\gg S_{\mathrm{AA}}\forall A\to X\;\text{is novel}$$

where X is the unknown class or antibiotic and S_AA_ is called the novelty threshold of known class A (Equation 3).

## Data Availability

The raw data supporting the conclusions of this article will be made available by the authors, without undue reservation.
