# Supplementary material for: HoloMoA: a holography and deep learning tool for the identification of antimicrobial mechanisms of action and the detection of novel MoA
Source: Front Microbiol. 2025 Aug 22;16:1640252. doi: 10.3389/fmicb.2025.1640252 (PMC12411554; doi:10.3389/fmicb.2025.1640252)
Supplement: Supplementary file 1 [file Data_Sheet_1.pdf]

Supplementary Material

HoloMoA: a holography and deep learning tool for the identification of antimicrobial mechanisms of action (MoA) and the detection of novel MoA

Zohreh Sedaghat[1†], Benoît Courbon[1†], Héloise Botrel[1], Hélène Dugua[1], Pawel Tulinski[1], Laethitia Alibaud[1], Lucia Pagani[1], Derry Mercer[1], Cyril Guyard[1], Christophe Védrine[2], Sophie Dixneuf[1*]

[1]BIOASTER, 40 avenue Tony Garnier, 69007 Lyon, France

[2]BIOASTER, 28 Rue du Dr Roux, 75015 Paris, France

*** Correspondence:** Corresponding Author sophie.dixneuf@bioaster.org

† These authors contributed equally to this work and share first authorship

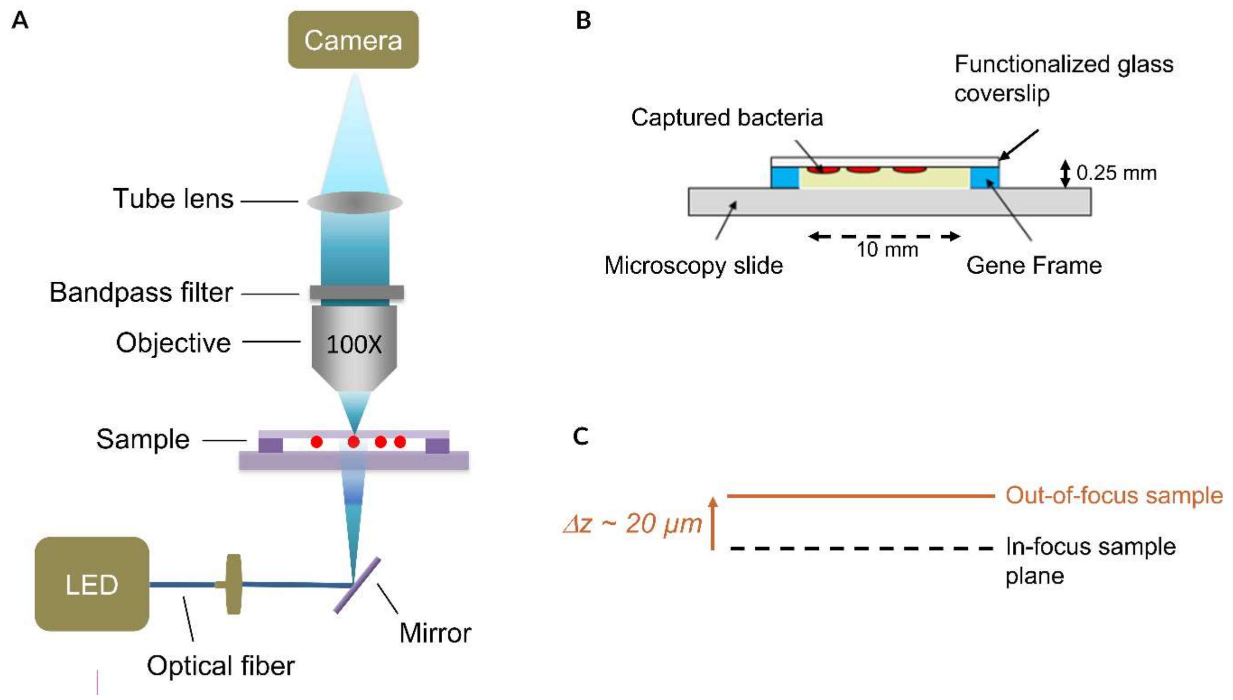

FIGURE S1 Experimental setup. (**A**) DIHM prototype. (**B**) Sample device. (**C**) Defocus condition for hologram recording.

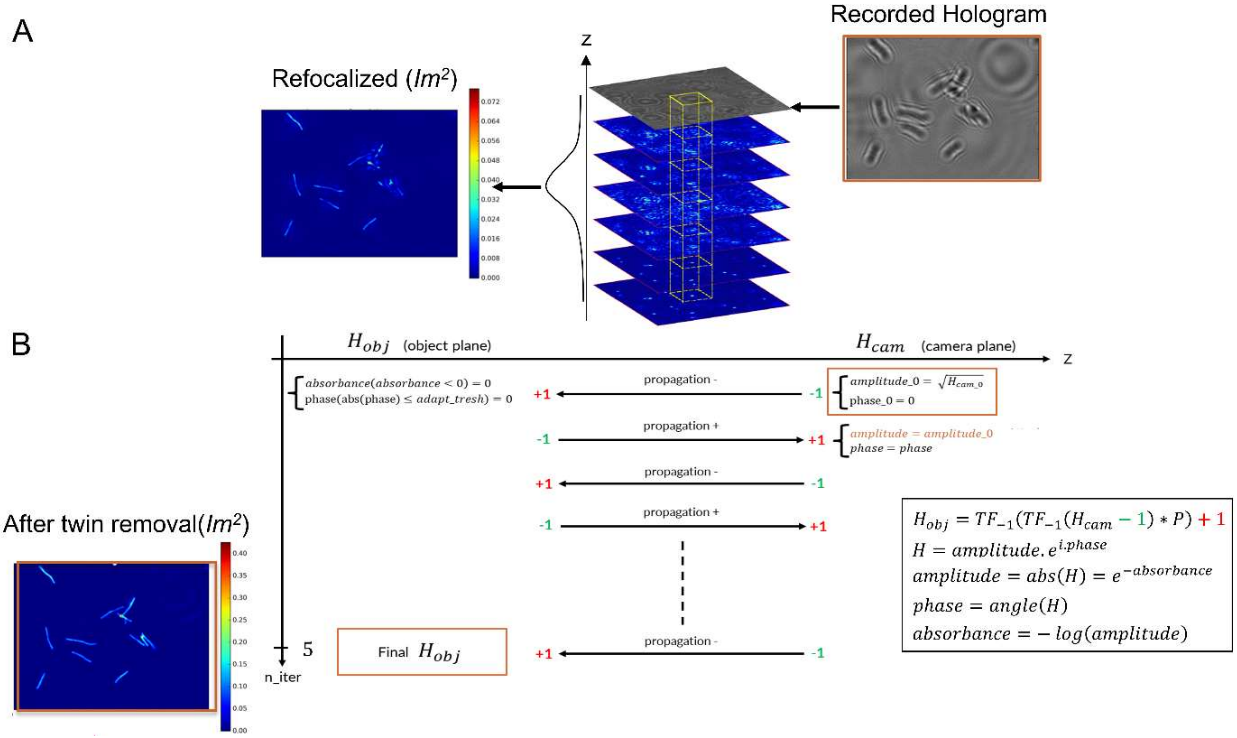

FIGURE S2 Hologram reconstruction principle, in 2 steps. (**A**) Digital refocalization. (**B**) Twin-image removal. H = complex wave field, TF = Fourier Transform, P = Rayleigh Sommerfeld propagator.

Incubation time	0 min	30min	60 min	90 min	120 min
Amoxicillin	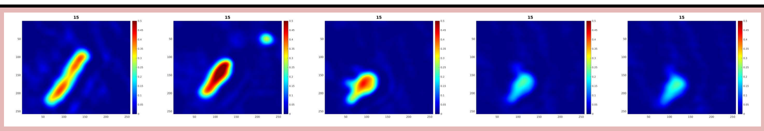				
Ampicillin	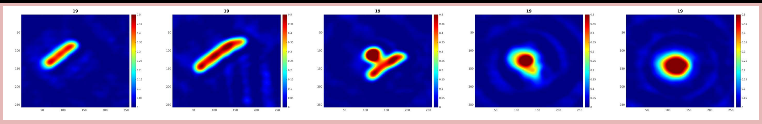				
Piperacilin	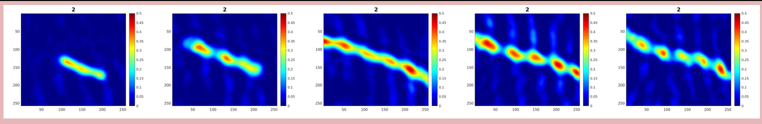				
Mecillinam	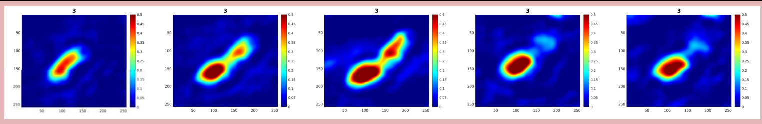				
Cefazolin	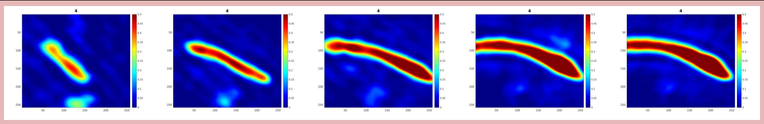				
Ceftazidime	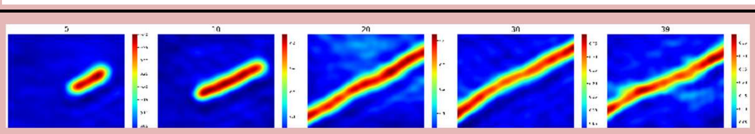				
Imipenem	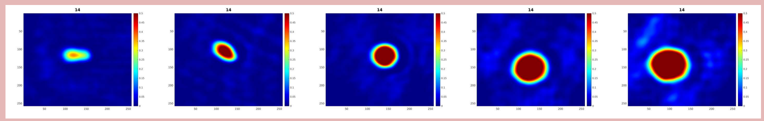				
Meropenem	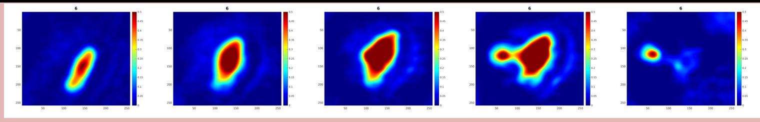				
Fosfomycin	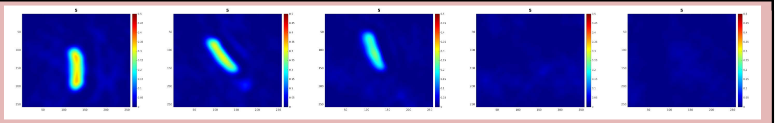				
Ciprofloxacin	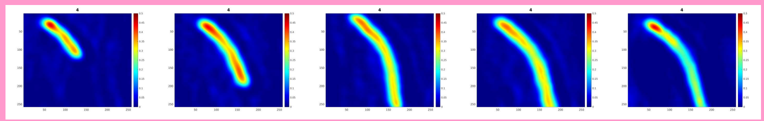				
Levofloxacin	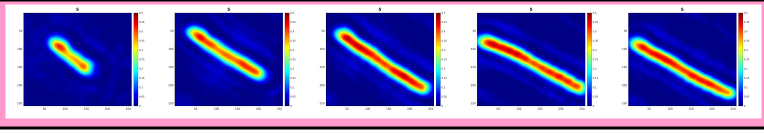				

Moxifloxacin	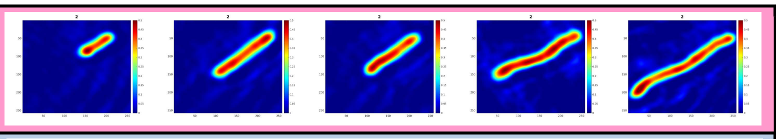
Rifampicin	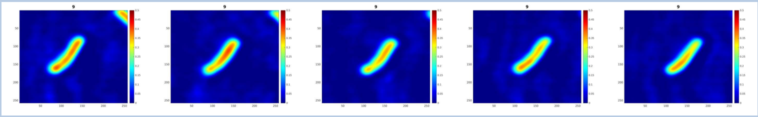
Rifapentin	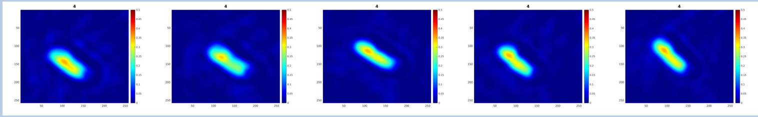
Kanamycin	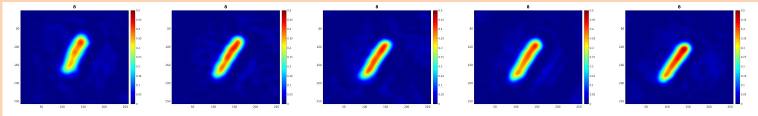
Tobramycine	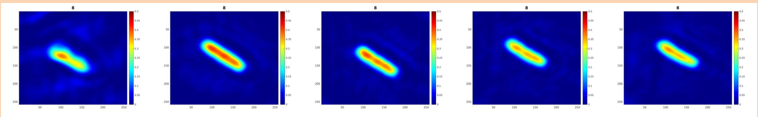
Tetracycline	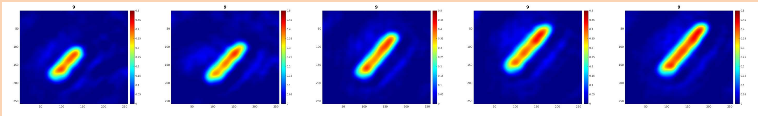
Lymecycline	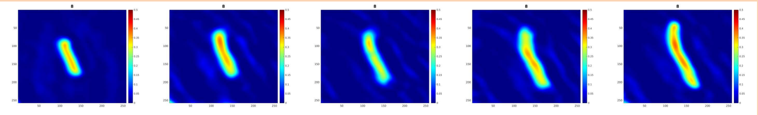
Chloramphenicol	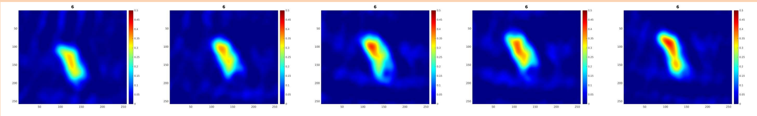
Thiamphenicol	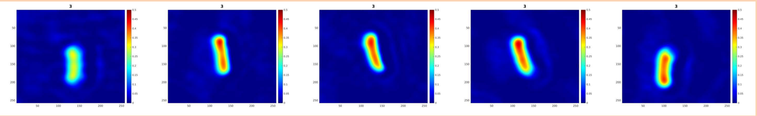
Mupirocin	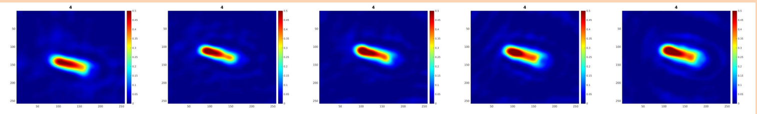
Colistin_MH2	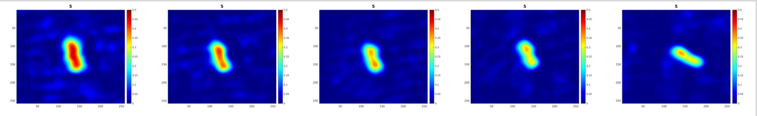
Trimethoprim-Sulfamethoxazole	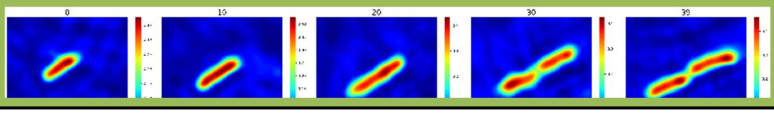

FIGURE S3 Examples of one dynamic patch for each molecule tested on *E. coli* ATCC 25922. Each patch shows a region of ~7x7μm^2. Here we show only one patch every 30 min.

(A)

Class	Precision	Recall	F1-score	Support
**Cell_Membrane**	0.80	0.88	0.84	96
**Cell_Wall**	0.87	0.83	0.85	481
**DNA**	0.73	0.64	0.68	170
**Protein**	0.73	0.79	0.76	387
**RNA**	0.74	0.68	0.71	251
**Untreated**	0.75	0.85	0.80	226
**Accuracy**	NA	NA	0.78	1611
**Macro average**	0.77	0.77	0.77	1611
**Weighted average**	0.78	0.78	0.78	1611

(B)

Class	Precision	Recall	F1-score	Support
**Cell_Membrane**	0.64	0.75	0.69	96
**Cell_Wall**	0.89	0.81	0.85	481
**DNA**	0.57	0.66	0.61	170
**Protein**	0.70	0.67	0.68	387
**RNA**	0.63	0.60	0.62	251
**Untreated**	0.73	0.82	0.77	226
**Accuracy**	NA	NA	0.73	1611
**Macro average**	0.69	0.72	0.70	1611
**Weighted average**	0.73	0.73	0.73	1611

FIGURE S4 Deep-learning-based classification metrics for different models (**A**) Patch-level classification, CNN3D model. (**B**) Patch-level classification, CRNN model. **Note**: Metrics are obtained using scikit-learn classification report, see method documentation for more details (https://scikit-learn.org/stable/modules/generated/sklearn.metrics.classification_report.html#sklearn.metrics.classification_report)

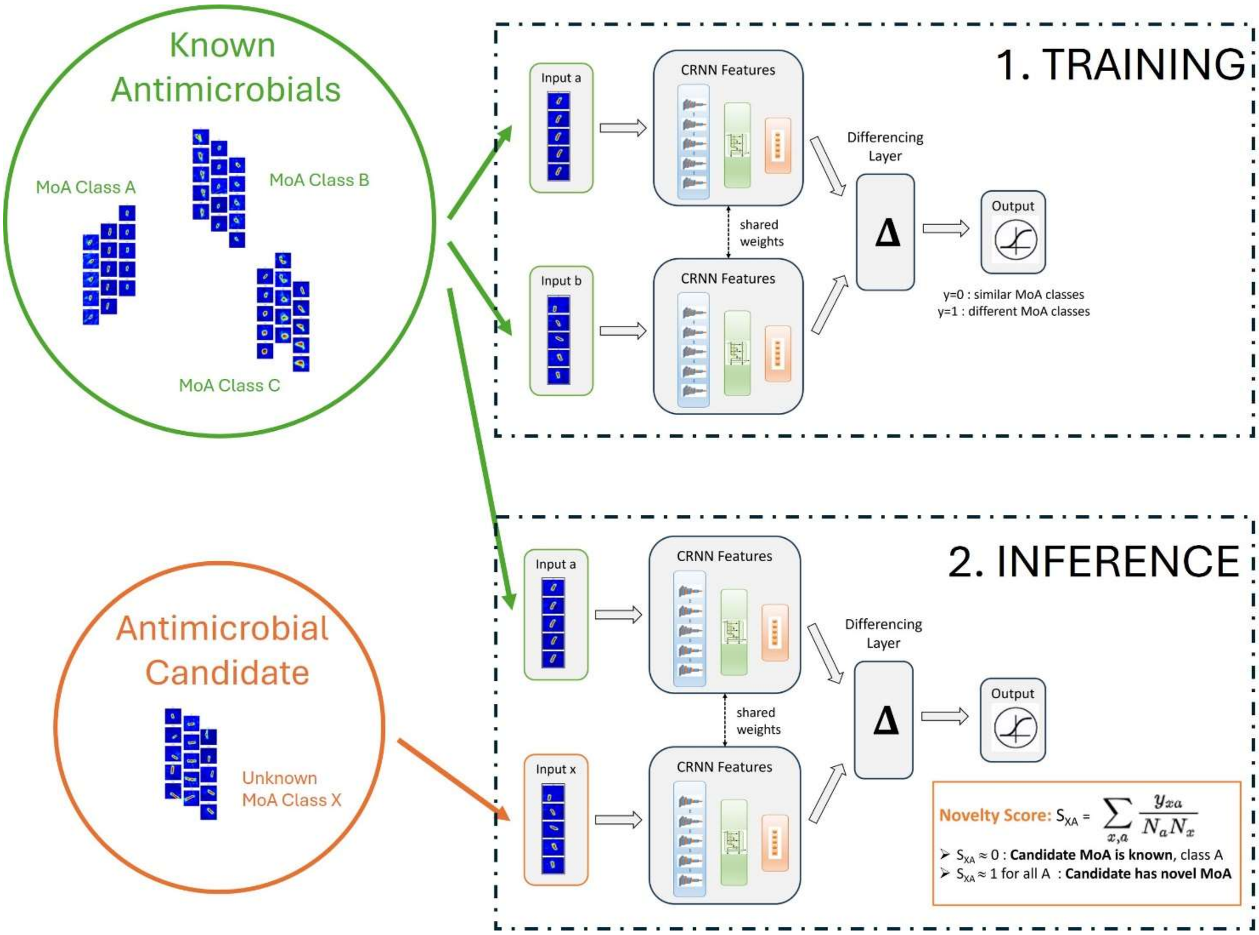

FIGURE S5 MoA novelty assessment pipeline.

(A)

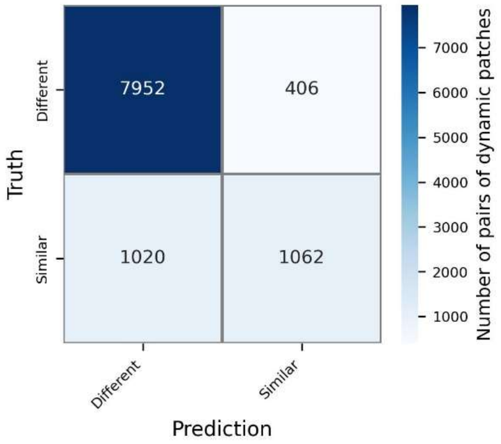

(B)

Class	Precision	Recall	F1-score	Support
**Different**	0.89	0.95	0.92	8358
**Similar**	0.72	0.51	0.60	2082
**Accuracy**	NA	NA	0.86	10440
**Macro average**	0.80	0.73	0.76	10440
**Weighted average**	0.85	0.86	0.85	10440

FIGURE S6 Results for the Deep-learning-based (sRCNN) classification of pairs of dynamic patches associated to similar or different MoA classes. (**A**) Confusion matrix. (**B**) Performance metrics.

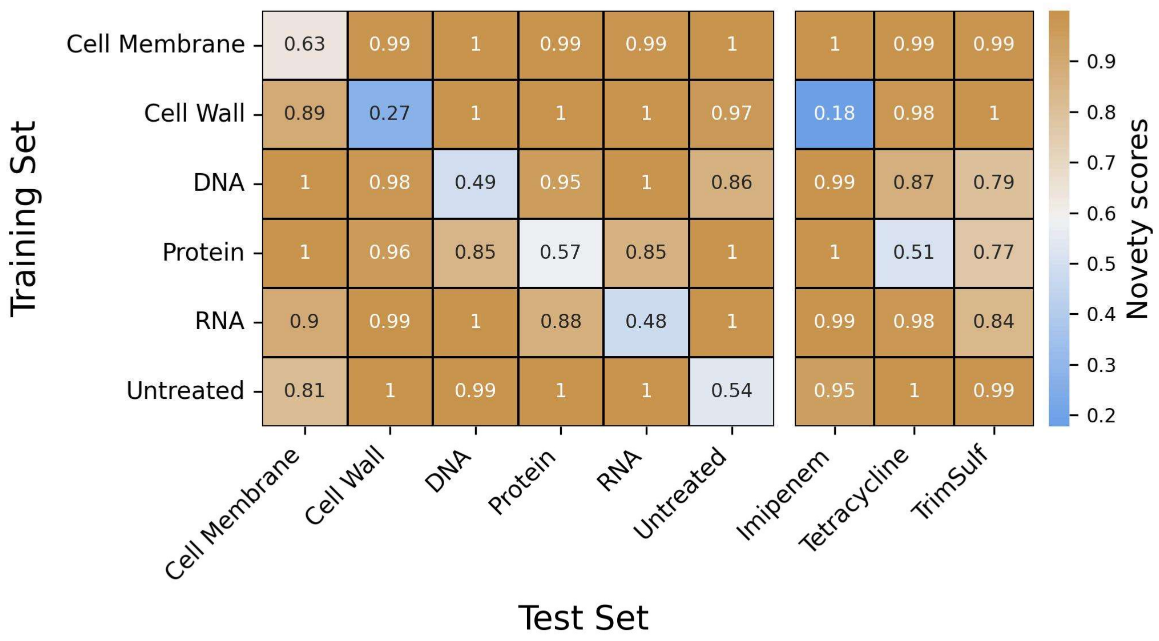

FIGURE S7 Novelty scores computed for test MoA classes or antibiotics with respect to training (i.e. known) MoA classes, based on pre-trained Deep Learning model (sRCNN).

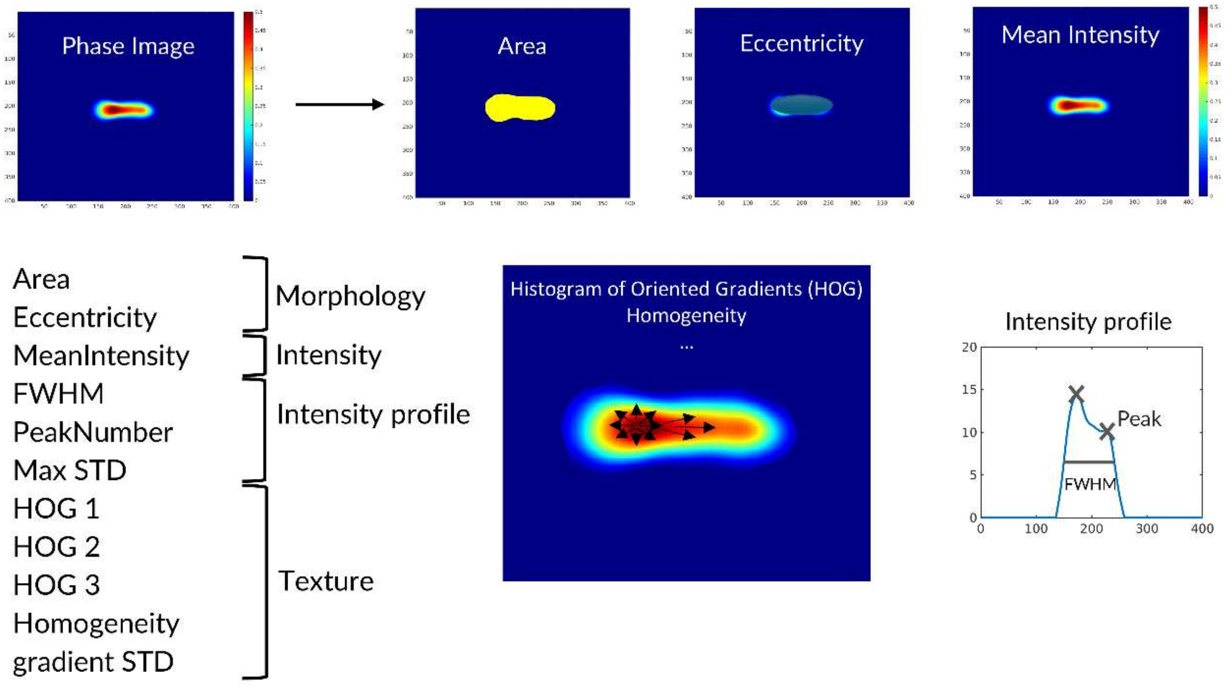

FIGURE S8. List and definition of hand-crafted (morphological/structural) features extracted from phase images of bacterial cells (**complementary data analysis, not used in DL-based analysis described in main manuscript**). **Note**: The HoloMoA technology relies on Deep Learning and does not involve hand-crafted feature extraction (i.e. the DL inputs are the dynamic patches, straightforwardly).

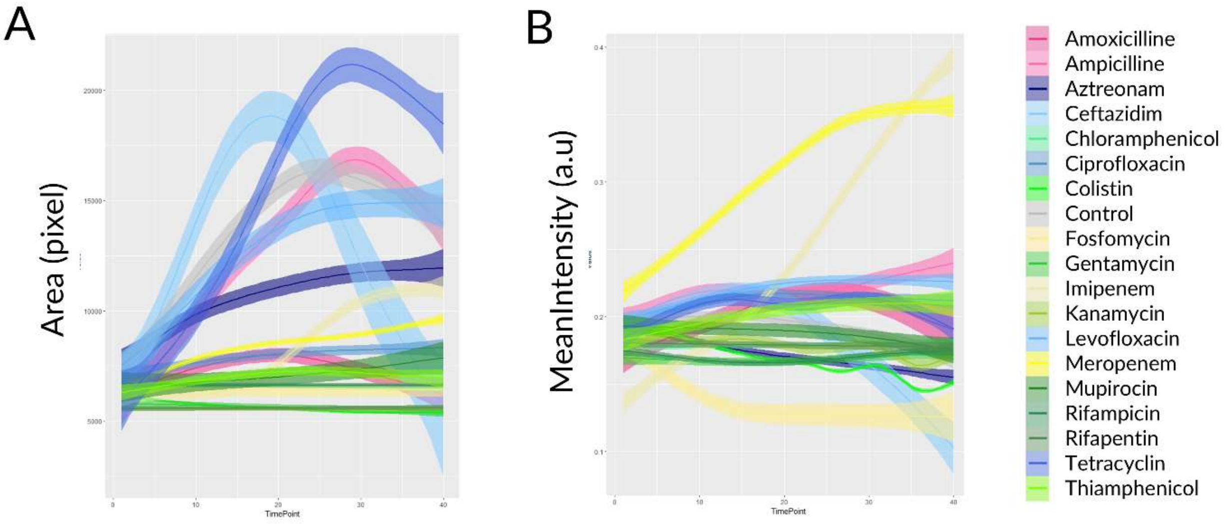

FIGURE S9 Examples of hand-crafted features extracted (as described in FIG S7) from dynamic phase patches of ~10 ATCC 25922 bacterial cells treated with 18 different antibiotics, plotted as a function of the duration of incubation (**complementary data analysis, not used in DL-based analysis described in main manuscript**). (**A**) Mean area of the bacteria and standard deviation; (**B**) Mean phase intensity of the bacteria and standard deviation. **Note**: The X axis shows the time-point stamps, where the last time-point (i.e. "40"), corresponds to 120 min of incubation. **Note**: The HoloMoA technology relies on Deep Learning and does not involve hand-crafted feature extraction (i.e. the DL inputs are the dynamic patches, straightforwardly).

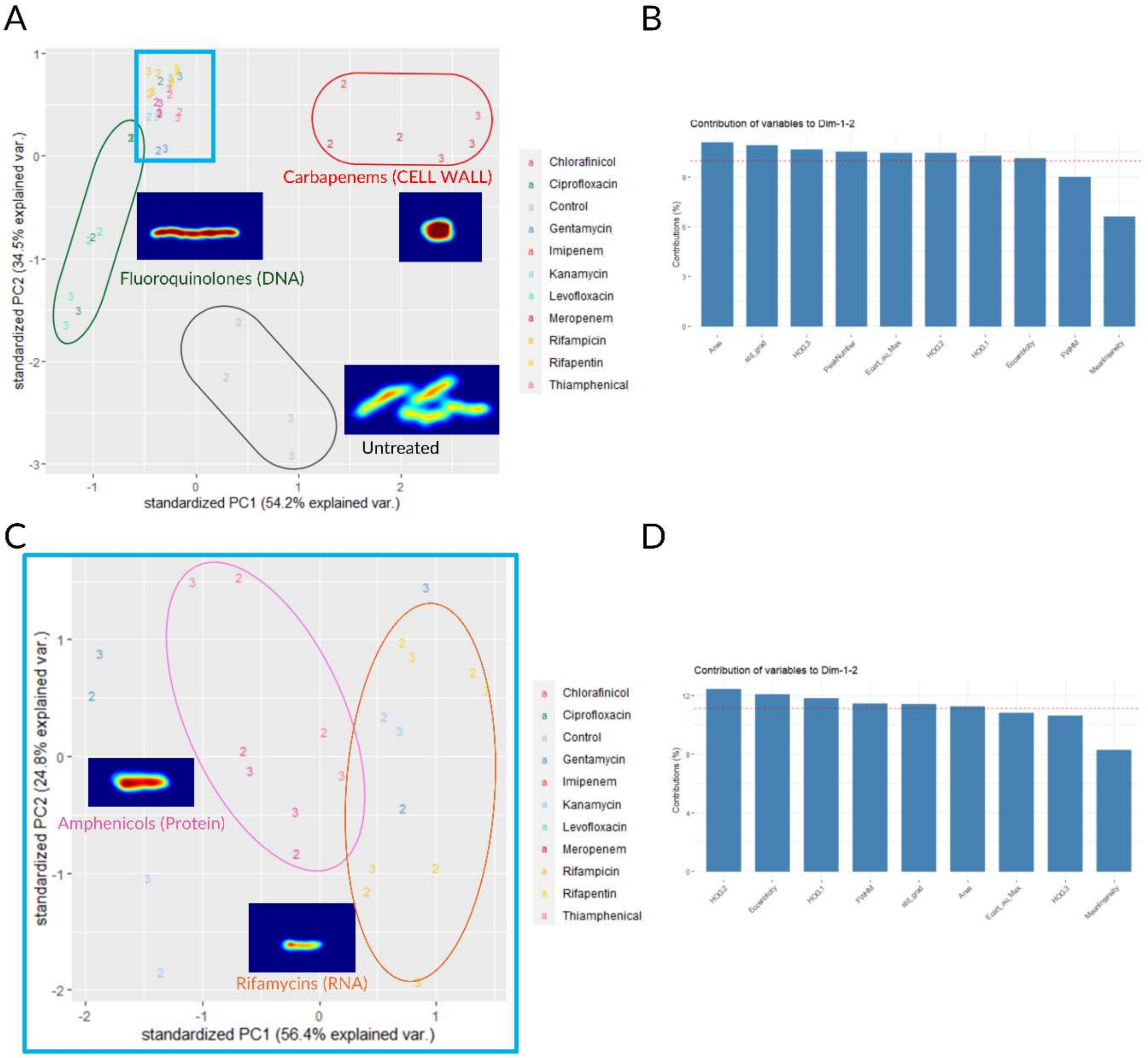

FIGURE S10. Principal Component Analysis (PCA) of 10 hand-crafted features from dynamical patches (as described in FIG8) of *E. coli* ATCC 25922 treated with different antibiotics (**complementary data analysis, not used in DL-based analysis described in main manuscript**). "2" stands for the median feature over 30-60 min, while "3" stands for the median feature over 60-90 min. (**A**) PC1 versus PC2 scores obtained when analyzing the features for 10 molecules and the untreated control. (**B**) Contribution of each feature to PC1 and PC2 dimensions linked to the A subplot, showing that both morphological and phase-related information contribute to differentiating the chemical classes. (**C**) PC1 versus PC2 scores obtained when analyzing the features for 6 molecules (amphenicols, rifamycins, aminoglycosides) and the untreated control. (**D**) Contribution of each feature to PC1 and PC2 dimensions linked to the C subplot, showing that both morphological and phase-related information contribute differentiating the chemical classes. **Note**: The HoloMoA technology relies on Deep Learning and does not involve hand-crafted feature extraction nor PCA analyses (i.e. the DL inputs are the dynamic patches, straightforwardly).

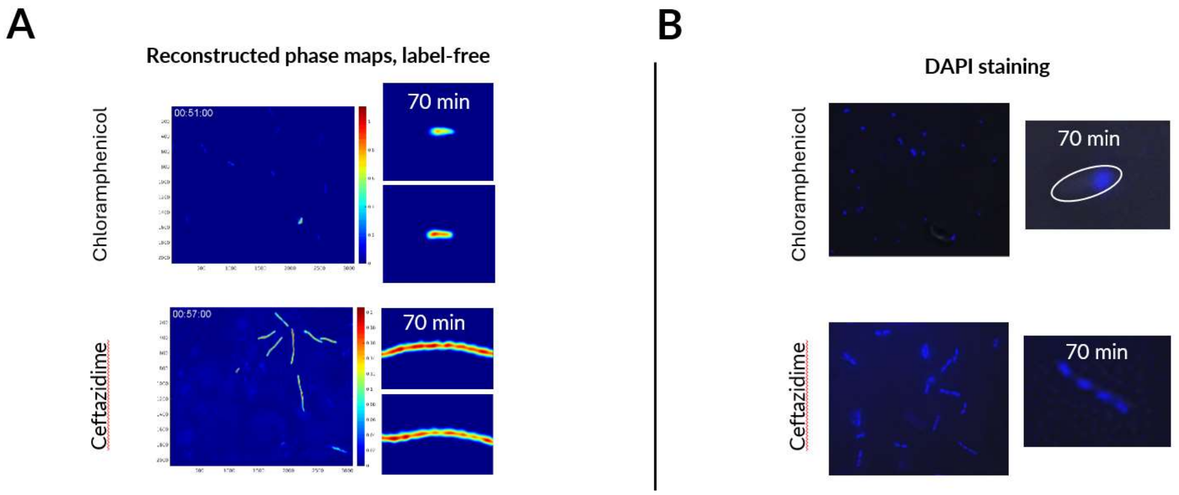

FIGURE S11 Comparison of DIHM and widefield epifluorescence microscopy **(side experiment, not described)** of *E. coli* ATCC 25922 incubated for 2h with chloramphenicol or ceftazidime. (**A**) time-lapse phase images (as described in the manuscript); (**B**) end-point fluorescence images (DAPI staining for DNA visualization).

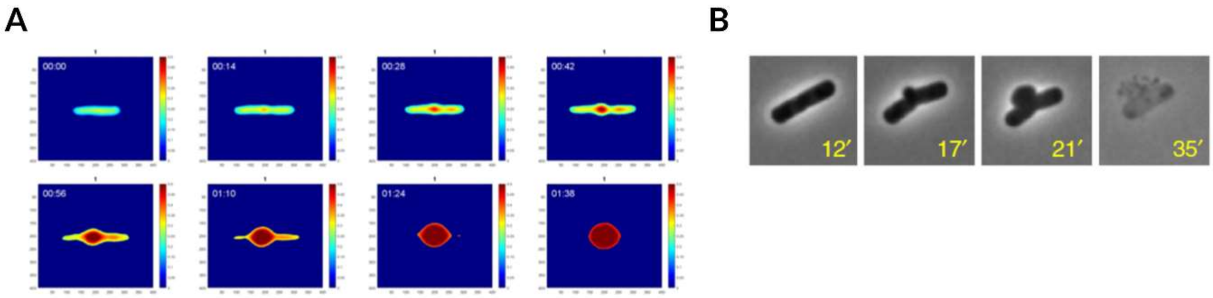

FIGURE S12 Comparing time-lapse Holographic images and time-lapse Phase contrast images acquired during incubation of two *E. coli* with beta-lactam antibiotics. (**A**) *E. coli* ATCC 25922 treated with amoxicillin (i.e. a penicillin) in MHB, observed with DIHM with x86 magnification[1]. (**B**) *E. coli* (from the Keio Knockout Collection) treated with cefsulodin (i.e. a cephalosporin) in LB, observed with phase-contrast microscopy with x40 magnification, extracted from Zahir et al[2].

[1] This work

[2] Zahir T, Camacho R, Vitale R, Ruckebusch C, Hofkens J, Fauvart M, Michiels J. 2019. High-throughput time-resolved morphology screening in bacteria reveals phenotypic responses to antibiotics. Commun Biol. 2:269.

(A)

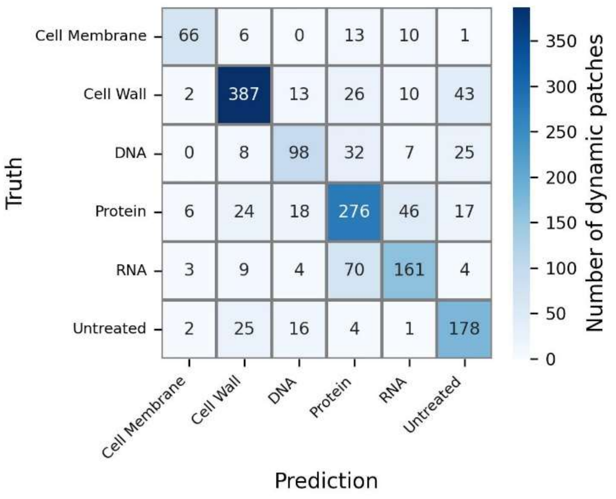

(B)

Class	Precision	Recall	F1-score	Support
**Cell_Membrane**	0.84	0.69	0.75	96
**Cell_Wall**	0.84	0.8	0.82	481
**DNA**	0.66	0.58	0.61	170
**Protein**	0.66	0.71	0.68	387
**RNA**	0.69	0.64	0.66	251
**Untreated**	0.66	0.79	0.72	226
**Accuracy**	NA	NA	0.72	1611
**Macro average**	0.72	0.7	0.71	1611
**Weighted average**	0.73	0.72	0.72	1611

FIGURE S13 Results for the Deep-learning-based classification of dynamic patches when using 5 time points (30 minutes time-lapse) (**A**) Confusion matrix, patch-level. (**B**) Performance metrics, patch-level.

(A)

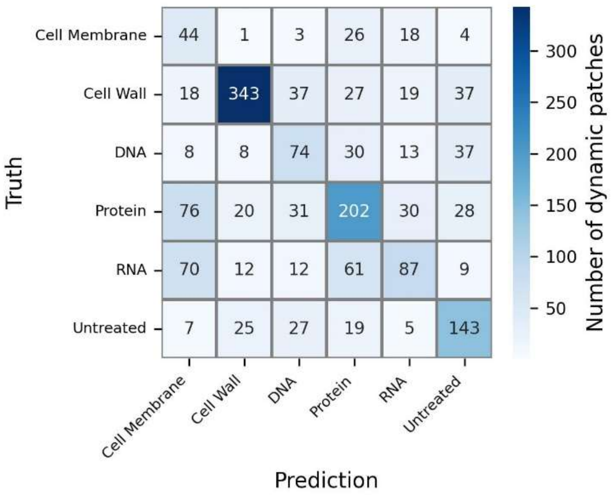

(B)

Class	Precision	Recall	F1-score	Support
**Cell_Membrane**	0.20	0.46	0.28	96
**Cell_Wall**	0.84	0.71	0.77	481
**DNA**	0.40	0.44	0.42	170
**Protein**	0.55	0.52	0.54	387
**RNA**	0.51	0.35	0.41	251
**Untreated**	0.55	0.63	0.59	226
**Accuracy**	NA	NA	0.55	1611
**Macro average**	0.51	0.52	0.50	1611
**Weighted average**	0.59	0.55	0.57	1611

FIGURE S14 Results for the Deep-learning-based classification of dynamic patches when using 1 time point (after 1 hour) (**A**) Confusion matrix, patch-level. (**B**) Performance metrics, patch-level.

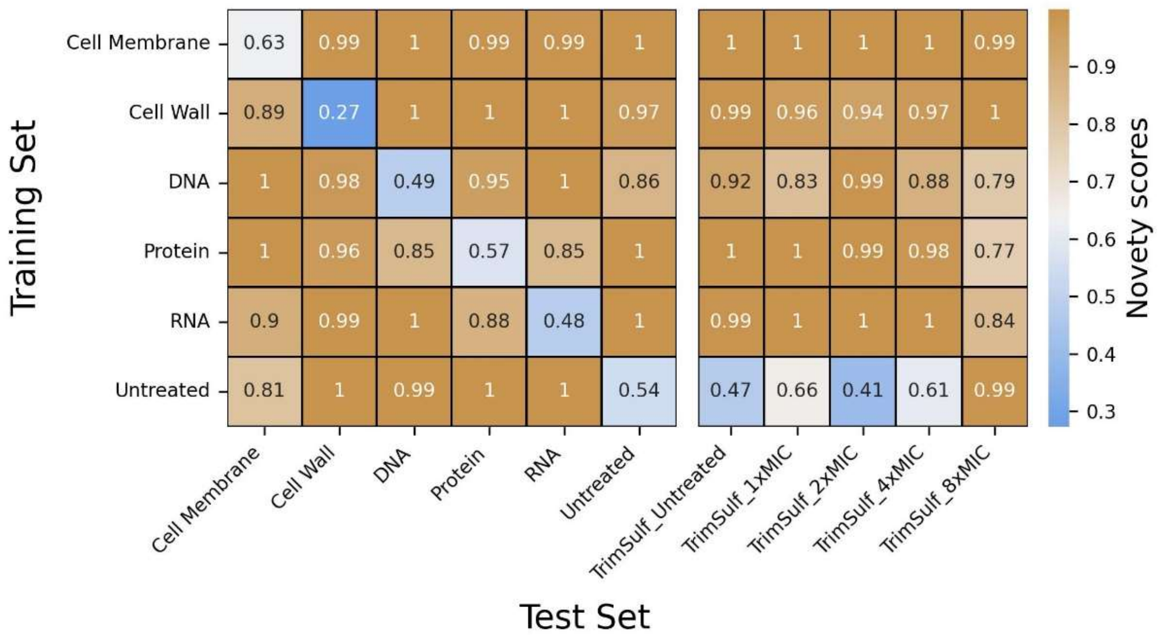

FIGURE S15 Novelty scores computed for test MoA classes and the trim-sulf candidate with different concentrations, with respect to training (i.e. known) MoA classes, based on pre-trained Deep Learning model (sRCNN).

TABLE S1 List of antibiotic molecules, solvent, measured MIC in MHB and CAMHB, and CLSI acceptance range.

Chemical class	Chemical Subclass	Molecule	MIC – MHB (µg/mL)	MIC – CAMHB (µg/mL)	MIC-CAMHB CLSI range (µg/mL)
β-lactam	Penicillin	Amoxicillin	4	8	Not available
		Ampicillin	8	4	2-8
		Piperacillin	4	4	1-4
		Mecillinam	0.031	0.125	0.03-0.25
	Carbapenem	Imipenem	0.25	Not measured	0.06-0.5
		Meropenem	≤0.125[a]	Not measured	0.008-0.06
	Cephalosporin	Cefazolin	4	4	1-4
		Ceftazidime	0.5	Not measured	0.06-0.5
Fosfomycin	Fosfomycin	Fosfomycin	4	Not measured	0.5-2
Quinolone	Fluoroquinolone	Ciprofloxacin	≤0.125[a]	Not measured	0.004-0.016
		Levofloxacin	≤0.125[a]	Not measured	0.008-0.06
		Moxifloxacin	0.062	0.062	0.008-0.06
Ansamycin	Rifamycin	Rifampicin	4	Not measured	4-16
		Rifapentine	4	Not measured	Not available
Aminoglycoside	Aminoglycoside	Kanamycin	4	4	1-4
		Tobramycin	2	2	0.25-1
Tetracycline	Tetracycline	Tetracycline	0.5	1	0.5-2
		Lymecycline	1	2	Not available
Amphenicol	Amphenicol	Chloramphenicol	4	Not measured	2-8
		Thiamphenicol	64[b]	Not measured	Not available
Mupirocin	Mupirocin	Mupirocin	8	Not measured	Not available
Polypeptide	Polymyxin	Colistin	Not measured	2	0.25-2[c]
Anisole-Sulfonamide		Trimethoprim/ Sulfamethoxazole (1:19)	4/76	1/19	≤0.5/9.5

[a]For these molecules, 0.125 µg/mL was used for HoloMoA analysis.
[b]Thiamphenicol was not fully solubilized.
[c]No MIC range was indicated in the last CLSI version, but, since experiments started in 2021, earlier CLSI versions were considered.

TABLE S2 Deep-learning-based classification results after patch prediction aggregation for the untreated class and for 5 known MoA classes: Cell Wall synthesis inhibitors, Cell Membrane inhibitors, Proteins synthesis inhibitors, DNA synthesis inhibitors and RNA synthesis inhibitors. (**A**) Aggregation at sample level, CNN3D model. (**B**) Aggregation at antibiotic level, CNN3D model. (**C**) Aggregation at sample level, CRNN model. (**D**) Aggregation at antibiotic level, CRNN model.

(A)

Antibiotic	Replicate	Sample True Class	Sample Predicted Class	Patch Accuracy	Majority Vote
Amdinocillin	Rep1	Cell Wall	Cell Wall	0.96	True
Meropenem	Rep2	Cell Wall	Cell Wall	0.93	True
Meropenem	Rep1	Cell Wall	Cell Wall	0.93	True
Amdinocillin	Rep2	Cell Wall	Cell Wall	0.92	True
Ampicillin	Rep1	Cell Wall	Cell Wall	0.92	True
Untreated	Rep1	Untreated	Untreated	0.90	True
Amoxicillin	Rep1	Cell Wall	Cell Wall	0.90	True
Thiamphenicol	Rep2	Protein	Protein	0.90	True
Amoxicillin	Rep3	Cell Wall	Cell Wall	0.90	True
Untreated	Rep3	Untreated	Untreated	0.89	True
Lymecycline	Rep3	Protein	Protein	0.88	True
Fosfomycin	Rep1	Cell Wall	Cell Wall	0.88	True
Chloramphenicol	Rep1	Protein	Protein	0.88	True
Colistin	Rep1	Cell Membrane	Cell Membrane	0.88	True
Chloramphenicol	Rep2	Protein	Protein	0.86	True
Tobramycin	Rep2	Protein	Protein	0.86	True
Rifapentine	Rep2	RNA	RNA	0.83	True
Untreated	Rep2	Untreated	Untreated	0.82	True
Untreated	Rep2	Untreated	Untreated	0.82	True
Kanamycin	Rep3	Protein	Protein	0.82	True
Fosfomycin	Rep2	Cell Wall	Cell Wall	0.81	True
Levofloxacin	Rep1	DNA	DNA	0.81	True
Tobramycin	Rep1	Protein	Protein	0.80	True
Untreated	Rep1	Untreated	Untreated	0.79	True
Ampicillin	Rep3	Cell Wall	Cell Wall	0.78	True
Levofloxacin	Rep2	DNA	DNA	0.76	True
Kanamycin	Rep1	Protein	Protein	0.75	True
Mupirocin	Rep1	Protein	Protein	0.74	True
Thiamphenicol	Rep1	Protein	Protein	0.72	True
Rifampicin	Rep2	RNA	RNA	0.67	True
Lymecycline	Rep1	Protein	Protein	0.61	True
Mupirocin	Rep2	Protein	Protein	0.57	True
Ampicillin	Rep2	Cell Wall	Cell Wall	0.55	True
Cefazolin	Rep2	Cell Wall	Cell Wall	0.50	True
Ciprofloxacin	Rep2	DNA	DNA	0.50	True

Moxifloxacin	Rep3	DNA	DNA	0.43	True
Piperacillin	Rep3	Cell Wall	Untreated	0.43	False
Piperacillin	Rep2	Cell Wall	Untreated	0.40	False
Rifampicin	Rep1	RNA	RNA	0.40	True
Rifapentine	Rep1	RNA	RNA	0.36	True
Ceftazidime	Rep1	Cell Wall	Untreated	0.33	False
Ceftazidime	Rep2	Cell Wall	Untreated	0.23	False

(B)

**Antibiotic**	**Antibiotic True Class**	**Antibiotic Predicted Class**	**Patch Accuracy**	**Majority Vote**
Amdinocillin	Cell Wall	Cell Wall	0.93	True
Meropenem	Cell Wall	Cell Wall	0.93	True
Amoxicillin	Cell Wall	Cell Wall	0.90	True
Colistin	Cell Membrane	Cell Membrane	0.88	True
Chloramphenicol	Protein	Protein	0.87	True
Fosfomycin	Cell Wall	Cell Wall	0.86	True
Untreated	Untreated	Untreated	0.85	True
Tobramycin	Protein	Protein	0.85	True
Lymecycline	Protein	Protein	0.81	True
Kanamycin	Protein	Protein	0.80	True
Thiamphenicol	Protein	Protein	0.79	True
Levofloxacin	DNA	DNA	0.77	True
Ampicillin	Cell Wall	Cell Wall	0.75	True
Rifapentine	RNA	RNA	0.74	True
Mupirocin	Protein	Protein	0.62	True
Rifampicin	RNA	RNA	0.52	True
Ciprofloxacin	DNA	DNA	0.50	True
Cefazolin	Cell Wall	Cell Wall	0.50	True
Moxifloxacin	DNA	x	0.43	False
Piperacillin	Cell Wall	x	0.42	False
Ceftazidime	Cell Wall	Untreated	0.26	False

(C)

**Antibiotic**	**Replicate**	**Sample True Class**	**Sample Predicted Class**	**Patch Accuracy**	**Majority Vote**
Meropenem	Rep2	Cell Wall	Cell Wall	0.95	True
Meropenem	Rep1	Cell Wall	Cell Wall	0.95	True
Fosfomycin	Rep2	Cell Wall	Cell Wall	0.94	True
Fosfomycin	Rep1	Cell Wall	Cell Wall	0.92	True
Ampicillin	Rep1	Cell Wall	Cell Wall	0.92	True
Untreated	Rep2	Untreated	Untreated	0.90	True
Amdinocillin	Rep1	Cell Wall	Cell Wall	0.88	True
Untreated	Rep3	Untreated	Untreated	0.86	True
Untreated	Rep1	Untreated	Untreated	0.85	True
Amoxicillin	Rep3	Cell Wall	Cell Wall	0.85	True
Kanamycin	Rep1	Protein	Protein	0.83	True

Amdinocillin	Rep2	Cell Wall	Cell Wall	0.83	True
Amoxicillin	Rep1	Cell Wall	Cell Wall	0.80	True
Lymecycline	Rep3	Protein	Protein	0.79	True
Untreated	Rep2	Untreated	Untreated	0.79	True
Thiamphenicol	Rep2	Protein	Protein	0.75	True
Cefazolin	Rep2	Cell Wall	Cell Wall	0.75	True
Colistin	Rep1	Cell Membrane	Cell Membrane	0.75	True
Ampicillin	Rep3	Cell Wall	Cell Wall	0.75	True
Ciprofloxacin	Rep2	DNA	DNA	0.73	True
Kanamycin	Rep3	Protein	Protein	0.72	True
Levofloxacin	Rep2	DNA	DNA	0.69	True
Mupirocin	Rep1	Protein	Protein	0.68	True
Levofloxacin	Rep1	DNA	DNA	0.67	True
Ceftazidime	Rep1	Cell Wall	Cell Wall	0.67	True
Rifapentine	Rep2	RNA	RNA	0.66	True
Untreated	Rep1	Untreated	Untreated	0.66	True
Thiamphenicol	Rep1	Protein	Protein	0.64	True
Chloramphenicol	Rep2	Protein	Protein	0.61	True
Lymecycline	Rep1	Protein	Protein	0.61	True
Rifampicin	Rep2	RNA	RNA	0.61	True
Rifampicin	Rep1	RNA	RNA	0.60	True
Tobramycin	Rep1	Protein	Protein	0.60	True
Tobramycin	Rep2	Protein	Protein	0.57	True
Chloramphenicol	Rep1	Protein	Protein	0.56	True
Moxifloxacin	Rep3	DNA	DNA	0.54	True
Ampicillin	Rep2	Cell Wall	Cell Wall	0.50	True
Mupirocin	Rep2	Protein	Protein	0.48	True
Ceftazidime	Rep2	Cell Wall	Cell Wall	0.38	True
Rifapentine	Rep1	RNA	RNA	0.33	True
Piperacillin	Rep2	Cell Wall	DNA	0.20	False
Piperacillin	Rep3	Cell Wall	DNA	0.14	False

(D)

Antibiotic	Antibiotic True Class	Antibiotic Predicted Class	Patch Accuracy	Majority Vote
Meropenem	Cell Wall	Cell Wall	0.95	True
Fosfomycin	Cell Wall	Cell Wall	0.93	True
Amdinocillin	Cell Wall	Cell Wall	0.84	True
Amoxicillin	Cell Wall	Cell Wall	0.84	True
Untreated	Untreated	Untreated	0.82	True
Colistin	Cell Membrane	Cell Membrane	0.75	True
Cefazolin	Cell Wall	Cell Wall	0.75	True
Kanamycin	Protein	Protein	0.75	True
Lymecycline	Protein	Protein	0.74	True
Ciprofloxacin	DNA	DNA	0.73	True

Ampicillin	Cell Wall	Cell Wall	0.72	True
Levofloxacin	DNA	DNA	0.68	True
Thiamphenicol	Protein	Protein	0.68	True
Rifampicin	RNA	RNA	0.60	True
Rifapentine	RNA	RNA	0.60	True
Chloramphenicol	Protein	Protein	0.59	True
Tobramycin	Protein	Protein	0.58	True
Moxifloxacin	DNA	DNA	0.54	True
Mupirocin	Protein	Protein	0.54	True
Ceftazidime	Cell Wall	Cell Wall	0.47	True
Piperacillin	Cell Wall	DNA	0.16	False

TABLE S3. Hyperparameters range for CRNN (**A**) and CNN3D (**B**) considered for optimization and their best value obtained.

(A)

Hyperparameter	Range	Best value
Learning rate	[1e-6, 1e-2]	6.00E-05
Number of convolutional kernels per layer	[[32,64], [32,64,128], [32,64,128,256], [64,128,256,512]]	[32,64,128,256]
Convolutional kernel size in xy dimensions	[3, 10]	5
Pooling size in xy dimensions	[1, 3]	2
Recurrent layer type	[LSTM, GRU]	LSTM
Number of recurrent units	[128, 512]	512
Number of dense units per layer	[[128], [512], [512,256]]	[512, 256]
Dropout rate	[0, 0.5]	0.4

(B)

Hyperparameter	Range	Best value
Learning rate	[1e-6, 1e-2]	6.00E-05
Number of convolutional kernels per layer	[[32,64], [32,64,128], [32,64,128,256], [64,128,256,512]]	[32,64,128,256]
Convolutional kernel size in xy dimensions	[3, 10]	5
Convolutional kernel size in time dimension	[2, 8]	5
Pooling size in xy dimensions	[1, 3]	2
Pooling size in time dimension	[1, 3]	2
Number of dense units per layer	[[128], [512], [512,256]]	[512, 256]
Dropout rate	[0, 0.5]	0.4

TABLE S4. Classification accuracy at patch, sample and antibiotic levels for different number of time points

Number of time points	5<br>(1, 30, 60, 90, 120min)	1<br>(1h)
**Patch-level accuracy**	72%	55%
**Sample-level accuracy**	88%	83%
**Antibiotic-level accuracy**	86%	81%
